# The role of proinflammatory cytokines and CXC chemokines (CXCL1–CXCL16) in the progression of prostate cancer: insights on their therapeutic management

**DOI:** 10.1186/s11658-024-00591-9

**Published:** 2024-05-14

**Authors:** Amin Ullah, Wang Jiao, Bairong Shen

**Affiliations:** Joint Laboratory of Artificial Intelligence for Critical Care Medicine, Department of Critical Care Medicine and Institutes for Systems Genetics, Frontiers Science Center for Disease-Related Molecular Network, West China Hospital, Sichuan University, Chengdu, China

**Keywords:** Prostate cancer (PCa), Cytokines, CXC chemokines, Inflammation, Targeted therapies

## Abstract

Reproductive cancers are malignancies that develop in the reproductive organs. One of the leading cancers affecting the male reproductive system on a global scale is prostate cancer (PCa). The negative consequences of PCa metastases endure and are severe, significantly affecting mortality and life quality for those who are affected. The association between inflammation and PCa has captured interest for a while. Inflammatory cells, cytokines, CXC chemokines, signaling pathways, and other elements make up the tumor microenvironment (TME), which is characterized by inflammation. Inflammatory cytokines and CXC chemokines are especially crucial for PCa development and prognosis. Cytokines (interleukins) and CXC chemokines such as IL-1, IL-6, IL-7, IL-17, TGF-β, TNF-α, CXCL1–CXCL6, and CXCL8–CXCL16 are thought to be responsible for the pleiotropic effects of PCa, which include inflammation, progression, angiogenesis, leukocyte infiltration in advanced PCa, and therapeutic resistance. The inflammatory cytokine and CXC chemokines systems are also promising candidates for PCa suppression and immunotherapy. Therefore, the purpose of this work is to provide insight on how the spectra of inflammatory cytokines and CXC chemokines evolve as PCa develops and spreads. We also discussed recent developments in our awareness of the diverse molecular signaling pathways of these circulating cytokines and CXC chemokines, as well as their associated receptors, which may one day serve as PCa-targeted therapies. Moreover, the current status and potential of theranostic PCa therapies based on cytokines, CXC chemokines, and CXC receptors (CXCRs) are examined.

## Introduction

Cancers that develop in the reproductive organs are referred to as reproductive cancers [1]. PCa is the most typical kind of male reproductive cancer [2]. Prostate-specific antigen (PSA) concentrations in the blood are assessed during PCa [3]. Regarding the number of diagnoses among malignancies, PCa comes in fourth [2]. An estimated 1.41 million new instances of cancer are reported each year, making up 7.3% of all cancer diagnoses [2]. In addition, it causes 375,000 deaths annually, or 3.8% of all cancer-related deaths [2]. Reproductive malignancies have been the focus of considerable research due to their high incidence and death rates.

There is evidence that chronic inflammation, caused by hormones, chemicals, radiation, stress, infectious agents, or other environmental factors, plays a crucial role in PCa development. This notion is supported by inflammation being a known risk factor for the PCa. Researchers observed that there was a connection between persistent prostate inflammation and the development of PCa. This may be because the prostate is more susceptible to infection than other parts of the body. Additionally, these findings are clearly evident by the histological features associated with inflammation seen in prostate tissues [4]. Numerous epidemiological studies have shown the direct relationship between inflammatory genes and the risk of PCa and the antagonistic relationship between PCa and anti-inflammatory medications [5, 6]. Thus, one of the instructional mechanisms involved in the development of cancer is inflammation. Malignant cells spatiotemporally release cytokines and CXC chemokines, and leukocyte subtypes undergo cell trafficking toward the TME [7]. The complex procedure of cell recruitment implies different leukocyte subsets with the ability to promote or inhibit malignancy by directing immune cells to the sites of inflammation.

## Methods

The investigation for this review paper was conducted using Google Scholar, PubMed/Medline, and Web of Science. It had been scheduled to conduct the literature review between January and August of 2023. Table 1 provides further details in this regard.

**Table 1:** Summing up the search methodology

Elements	Description
The date or time of the investigation	January 2023–August 2023
Timeframe	1997–2023
Investigate the databases and other online resources	Google Scholar, PubMed/Medline, and Web of Science
Performed research on appropriate keywords	Prostate cancer, prostate adenocarcinoma, proinflammatory cytokines (interleukins), CXC chemokines ligands (CXCL1–CXCL16), and CXC receptors (CXCRs)
Criteria for inclusion and exclusion	Only original research and reviews written in English
Any applicable extra information	We evaluated data from in-depth investigations, reviews that have previously been written, original studies, and clinical and preclinical trials

## Proinflammatory cytokines and CXC chemokines

**Fig. 1:**
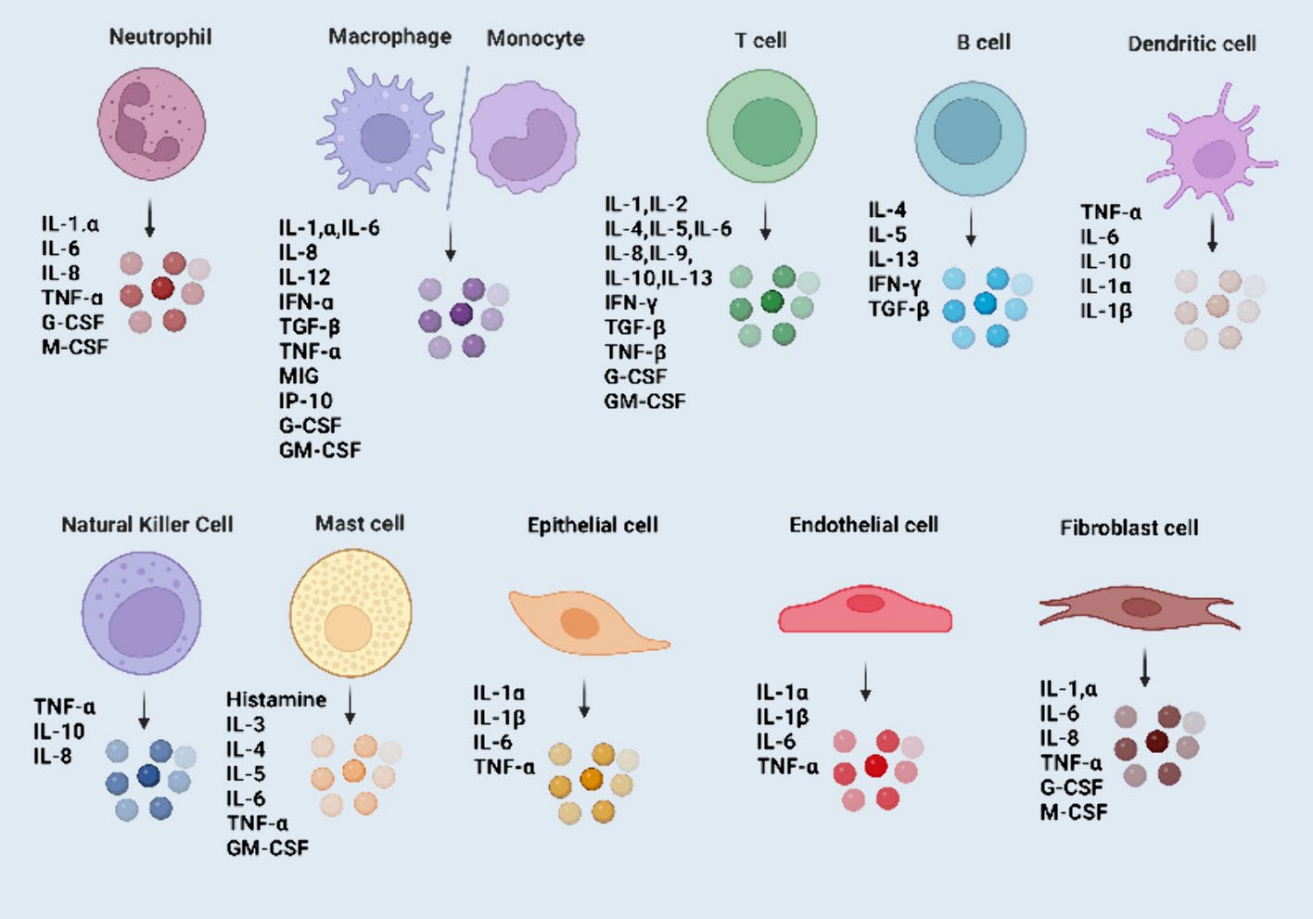
A schematic illustration of several cells that act as both major sources and targets of various proinflammatory cytokines, including interleukins, NK, TNF-α, mast cells, TGF-β, and GM-CSF, etc.

**Fig. 2:**
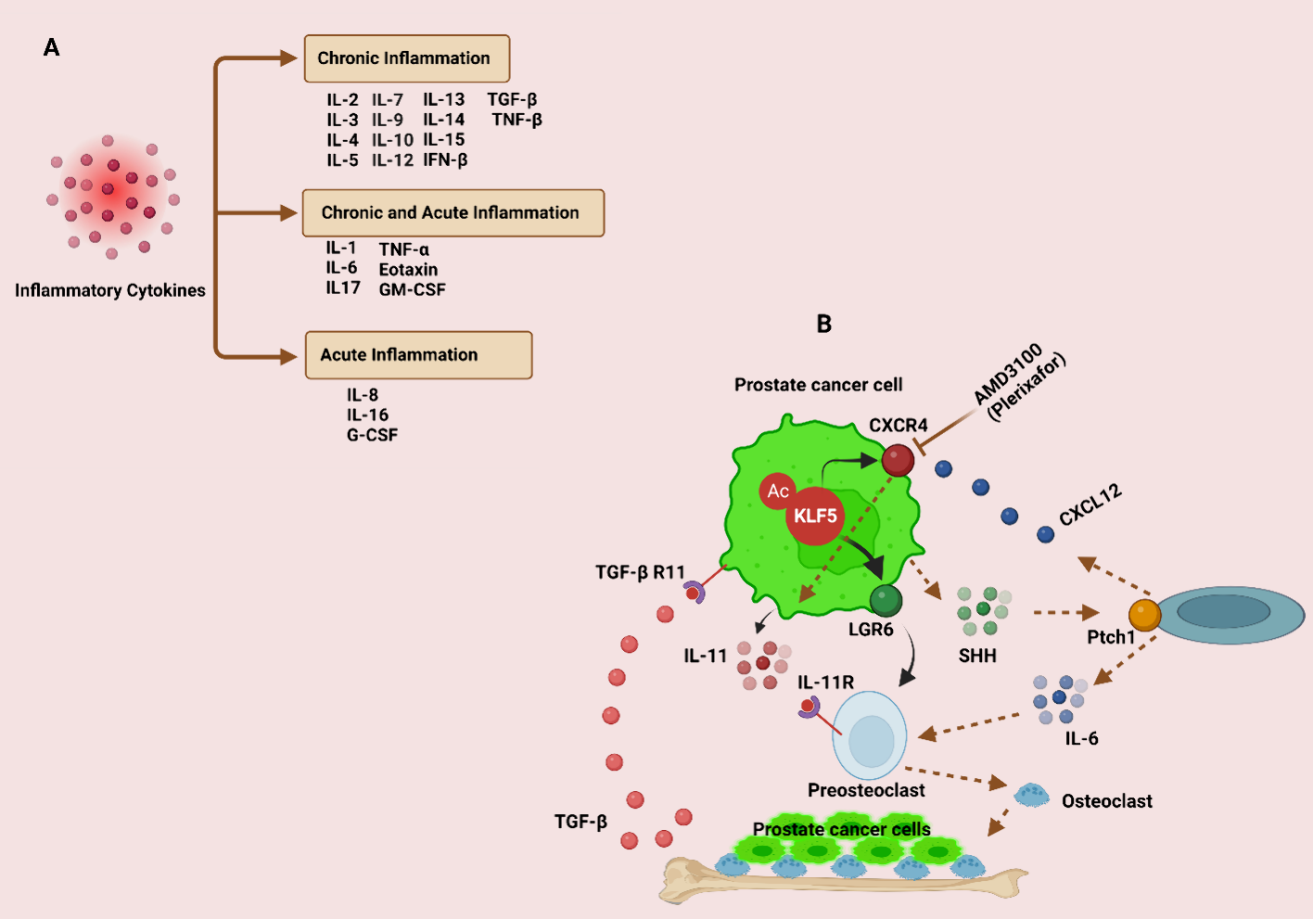
**A** List of cytokines that trigger both chronic and acute inflammation. **B** A diagram illustrating how acetylation of Krüppel‐like factor 5 (KLF5) promotes osteoclast formation by transcriptionally activating CXCR4, which in turn increases IL-11 production. This results in metastasis to the bones. Heavy acetylation of the KLF5 transcription factor in the bone microenvironment causes bone metastatic lesions by stimulating the CXCL12/CXCR4 chemokine axis and additional paracrine signaling pathways, such as those of IL-11 and soluble HH (SHH). This mechanism could have an impact on the detection and management of PCa bone metastases

Cytokines are small protein molecules that are released into the body and have a molecular weight of less than 40 kilodaltons. They are generated by practically every cell in the body to control and impact the immune response [8]. The secretion of proinflammatory cytokines will stimulate the production of further cytokines as well as the stimulation of immune cells [9]. Thus, when the phrase “cytokine storm” first appeared, it defined inflammation as the unexpected upregulation of an inflammatory process caused by the release of cytokines [10]. Recent studies, however, suggest that any immune response must include the simultaneous release of pro- and anti-inflammatory cytokines [11]. Multiple names are used to refer to cytokines, including interleukins (ILs), chemokines, and growth factors [12]. The so-called superfamilies that collectively make up cytokines rarely describe common genes but rather related structural features [13]. Moreover, numerous cell groups are capable of producing the same cytokine. Cytokines are pleiotropic because their actions vary depending on the cell they are targeting [12]. Furthermore, several cytokines may have the same effect and hence be unnecessary. However, they may have a synergistic impact. Finally, they may initiate signaling cascades, allowing even trace levels of protein to have disastrous implications [14]. Figures 1 and 2A provide a brief overview of several cells that express a variety of chronic and acutely induced inflammatory cytokines, as well as the cytokines involved in acute and chronic inflammatory responses [15, 16].

CXC chemokines are the main chemokine in terms of distribution and localization, and many of their genes have previously been defined [17]. Their bigger, serpentine G-protein-coupled receptors (GPCRs) regulate the several roles of CXC chemokines in organisms with multiple cells, including the distinctive cell movement [18]. We recently discussed the classification of CXC chemokines and their effects on immune surveillance in a number of inflammatory diseases, such as diabetes, nonalcoholic fatty liver disease, liver cancer, endometriosis, and polycystic ovary syndrome. These CXC chemokines function as chemotaxis, attracting immune cells and causing inflammation [19, 20]. We will thus have new opportunities for a better understanding of the role of CXC chemokines in the genesis of inflammatory disorders if we have a deeper knowledge of their potential mechanisms in inflammatory disorders and their management.

Interest in the associations between inflammation and PCa has developed with regard to the proinflammatory cytokines and CXC chemokines in PCa. It has been shown that chronic inflammatory conditions such as prostatitis raise the risk of PCa [21, 22]. Moreover, several recent studies have shown that these proinflammatory cytokines and CXC chemokines play a crucial role in the onset and progression of PCa [23–26]. By understanding how proinflammatory cytokines and CXC chemokines contribute to PCa development, we may be able to build a superior targeted therapy approach. Thus, our goal is to present an in-depth review of the most recent advancements in our knowledge of how proinflammatory cytokines and CXC chemokines influence the onset of PCa and the immune response to it. The complex interaction between proinflammatory cytokines and the CXC chemokines system is highlighted, and its potential use in PCa therapy is explored. Furthermore, the status and potential of theranostic PCa therapies based on cytokines, CXC chemokines, and CXCRs are examined.

### IL-1 and PCa

**Fig. 3:**
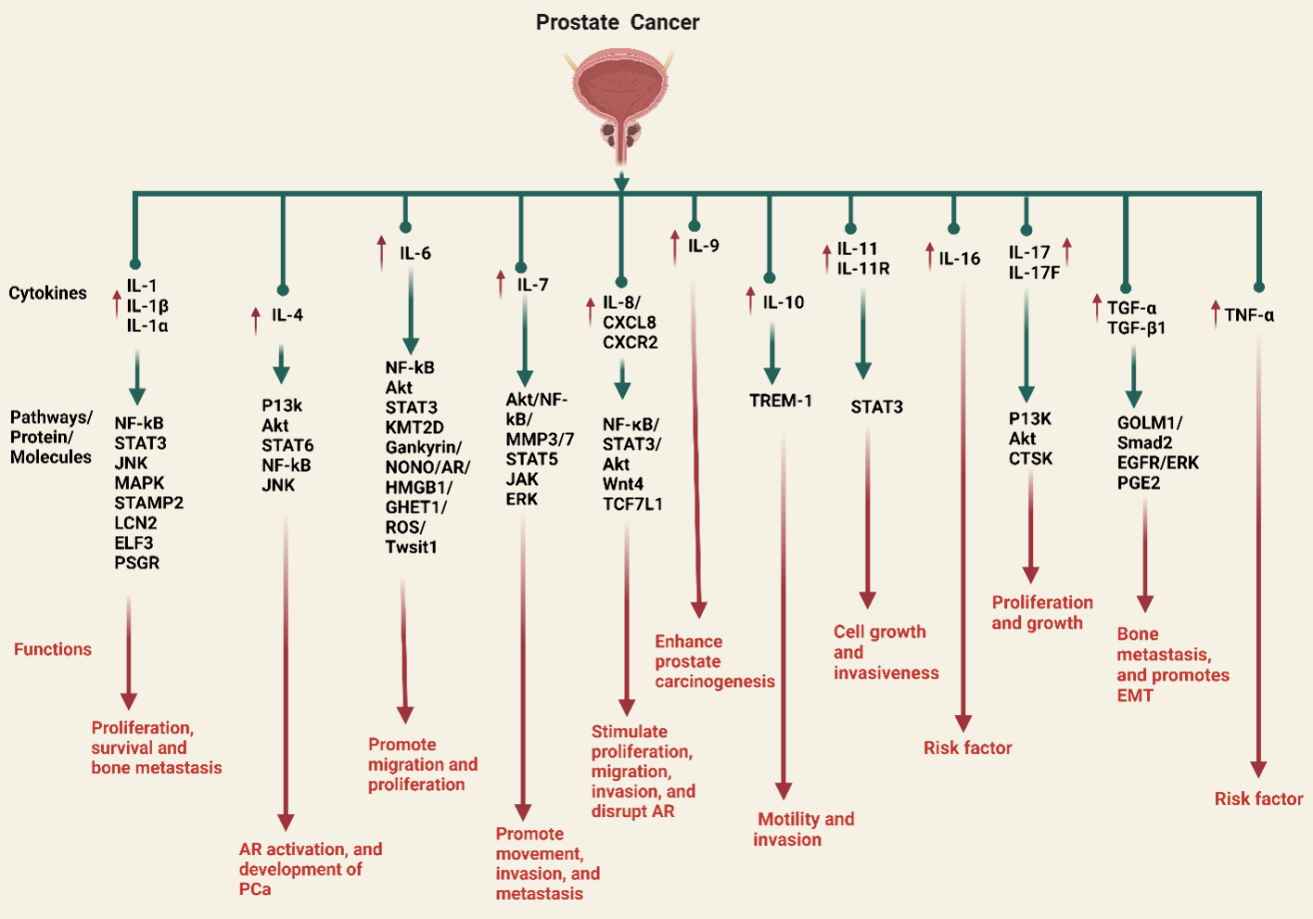
Proinflammatory cytokines (interleukins) play a role in PCa, as depicted in the illustration. IL-1 and the family’s members, including IL-α and IL-β, engage with several pathways, including NF-kB, STAT3, JNK, and MAPK, as well as proteins/molecules such as STAMP2, LCN2, ELF3, and PSGR, to promote PCa cell proliferation, survival, and bone metastasis. The PI3K/Akt/NF-kB pathways are responsible for IL-4-induced AR activation in PCa. Whereas the STAT6 pathway contributed to the development of PCa. IL-6 links with various signaling pathways, including NF-kB, STAT3, and Akt, as well as proteins and molecules such as KMT12D, Gankyrin/NONO/AR, HMGB1/GHRT1/Twsit1, and ROS, to facilitate the migration and proliferation of PCa cells. IL-7 stimulates MMP3 and MMP7 synthesis and activates the Akt/NF-kB pathway, which promotes PCa cell movement and invasion, while the STAT5, JAK, and ERK pathways promote EMT and metastasis. IL-8 stimulates PCa proliferation, migration, invasion, and defense against apoptosis via the NF-kB/STAT3/Akt pathway. IL-8/CXCR2 pathway activation and AR signaling disruption increase PCa NED and malignancy following Wnt4/TCF7L1 induction. IL-9 stimulates mast cell activation to enhance prostate carcinogenesis. AR signaling increases IL-10 and myeloid cell-1 (TREM-1) signaling on macrophages and improves PCa cell motility and invasion. Prostate microenvironment stromal cells’ paracrine IL-11 production via IL-11R–STAT3 signaling promotes PCa cell growth and invasiveness. IL-6 and TNF-α work as risk factors in PCa development. IL-17F triggered the PI3K/Akt signaling pathway to increase PCa cell malignancy, and the IL-17/CTSK axis controls PCa growth and proliferation. PCa membrane-bound TGF-α stimulates EGFR on osteoblasts during bone metastasis through cell-to-cell adhesion. Autocrine ERK signaling and PGE2 production by active EGFR increase bone development. The essential oncogene protein GOLM1 induces EMT in PCa by activating the TGF-β1/Smad2 signaling pathway. The up arrow (↑) symbol represents upregulation

Figure 3 illustrates the various ways that proinflammatory cytokines (interleukins) affect the onset and progression of PCa. The IL-1 family of cytokines presently consists of seven ligands with proinflammatory activity: IL-1α and IL-1β, IL-18, IL-33, IL-36α, IL-36β, and IL-36γ. It is generally known that these cytokines significantly affect how the innate and adaptive immune systems react. IL-1’s role in the emergence of malignancies is becoming more and more clear, as is the connection between inflammation and cancer [27]. It has been discovered that the expression of the six transmembrane protein of prostate 2 (STAMP2), which is important for the proliferation and survival of PCa cells, is modulated by inflammatory signals. Regarding this, STAMP2 can be synergistically induced by IL-1β through nuclear factor kappa B (NF-kB) and the production of signal transducer and activator of transcription 3 (STAT3). Androgen receptor (AR) signaling is not needed for this. Interestingly, PCa cells appear more vulnerable to cytokine treatment when STAMP2 is knocked down. Therefore, regulating STAMP2 through inflammatory cytokines may impact the progression of PCa (Fig. 3) [28]. These findings reveal that STAMP2 reacts to inflammatory signals and operates as a viability component for AR-positive PCa cells under these circumstances. Moreover, the levels of inflammatory mediator expression among the localized PCa tumors from 118 neoadjuvant-naive patients who had radical prostatectomy varied greatly, according to immunohistochemistry (IHC) analyses [29]; however, the majority of samples (> 94–95%) had, IL-1, and NF-kB expression, which is a recognized modulator of inflammatory reactions. Furthermore, early biochemical recurrence was linked to high IHC scores for IL-1 [29, 30]. According to these study data, PCa frequently exhibits inflammatory events and dysregulated cytokine (IL-1) production, which may play a role in etiology and disease progression.

A 25-kDa secreted glycoprotein known as lipocalin-2 (LCN2) serves both physiological and pathological purposes [31]. According to recent research, many signaling pathways, including p38, NF-kB, and c-Jun N-terminal kinase (JNK), were immediately activated following TNF treatment. Additionally, this research found that after 24 h of stimulation, IL-1 mRNA levels were considerably increased and induced LCN2. Mechanistically, the overexpression of LCN2 in PC-3 cells is directly mediated through the NF-kB pathway and the JNK signaling axis (Fig. 3) [32]. Thus, TNF-α can induce LCN2 protein expression and secretion in PC-3 cells. These data indicate strong evidence that besides the NF-kB pathway, the JNK signaling axis is responsible for the TNF-α-mediated LCN2 induction and is a potential therapeutic target for PCa patients, considering that LCN2 has been recognized as a tumor-promoting component in the disease. In addition, according to recent research, selenium, zinc, and iron affect the production of pro-inflammatory cytokine (IL-1), which in turn stimulate inflammation in PCa cells (LnCaP). They also interfere with the production of arachidonic and linoleic acid metabolites, which have proinflammatory effects and promote the growth and spread of PCa [33]. Abnormalities in Ca2^+^ signaling have a big impact on PCa development. According to Yu and others, the IL-1/NF-kB pathway is responsible for the formation of the endolysosomal ion channel MCOLN2 (Mucolipin-2) PCa [34]. The interesting prospect of using MCOLN2 as a therapeutic target in the treatment of PCa is highlighted by this work.

Meanwhile, IL-1α could hinder the growth of the tumor by causing PCa’s G0–G1 cell cycle arrest [35]. IL-1β was reported to induce Th1 and Th17 to strengthen the antitumor effect. IL-1β also exerts antitumor effects, which can prevent metastatic cells from colonizing in the metastatic place, thus inhibiting metastasis [27]. In addition, studies has shown that IL-1α and IL-1β act as tumor-specific Th1 mediators in the fight against cancer [36]. Regarding the protumor potential, it has been discovered that both IL-1α and IL-1β help with tumor angiogenesis and invasiveness as PCa develops [37]. It has been discovered that IL-1α and IL-1β might transform AR + PCa cells into AR-PCa cells, leading to castrate-resistant prostate cancer (CRPC) and therapy resistance [38]. According to research data, IL-1α may interact to create prostate-specific membrane antigen (PSMA) and PSA prostate clones [39].

Epithelium-specific ETS and ESE1 [or E74-like factor (ELF3)], two E26 transformation-specific (ETS) family members linked with PCa malignancy and a poor outcome for patients, may be triggered by IL-1β through the NF-kB pathway [40]. IL-1β may also stimulate the production of endothelin 1 (ET-1) and matrilysin 1, both of which have been linked to the development of PCa [41, 42]. Evidence suggests IL-1β plays a significant role in the development of PCa by activating the mitogen-activated protein kinase (MAPK) pathway, which triggers the induction of IL-8, increasing the potential for invasive growth and excessive proliferation of cells. Additionally, glucosamine consistently blocks the IL-1-mediated activation of MAPKs, thereby reducing the production of IL-8 (Fig. 3) [43].

One of the most commonly mutated or deleted genes in human PCa is the tumor suppressor gene phosphatase and tensin homolog (PTEN). DNA methylation sequencing and RNA sequencing investigations on the prostate-specific PTEN knockout (KO) mouse prostatic adenocarcinoma model revealed that PTEN knockout mice displayed upregulation of inflammatory genes and immune response pathways, such as IL-1, and NF-kB [44]. These data showed that PTEN loss promotes inflammation over time, notably through genes that regulate responses to inflammation and immune-mediated pathways. Moreover, NF-kB RELA (p65), recent research suggests RELA-independent mechanisms influence IL-1-mediated AR inhibition in LNCaP cells [45]. Comparing LNCaP xenograft tumors to culture cells revealed a transition from an androgen-responsive to an androgen-nonresponsive state. Inhibition of the AR and aryl-hydrocarbon pathways was discovered, and IL-1-mediated pathways were responsible for these modifications [46]. Moreover, according to Dahl et al., long-term IL-1 exposure favors the development of CRPC by encouraging PCa cell androgen and AR independence [47]. In response to short-term exposure to IL-1, MDA-PCa-2b cells suppress AR and AR activity, and they become resistant to long-term exposure. While LNCaP and MDA-PCa-2b cells have largely conserved biological and molecular responses to acute IL-1 signaling, including upregulation of NF-kB signaling and downregulation of cell proliferation, they have also evolved conserved and distinct molecular responses to chronic IL-1 signaling that may support or promote tumor progression [48].

A novel potential diagnostic and therapeutic target for PCa has been discovered as the prostate-specific G-protein-coupled receptor (PSGR) [49]. Based on recent research, PSGR may target IL-1β to modulate the MAKP and NF-kB signaling pathways involved in the formation of bone metastasis (Fig. 3) [50]. Furthermore, IL-1 has been linked to the stimulation of bone osteoclastogenesis. IL-1 was identified as one of the cytokines related to osteoclastogenesis and with associated metastasis-promoting potential using a SCID mouse metastatic model of PCa [51]. In chondrocytes, IL-1 induces apoptosis and the degradation of the cartilage matrix through miR-142-5p/RUNX2, which is purportedly accelerated by exosomal PCa gene expression marker 1 [52]. It provided a novel perspective on the genesis of osteoarthritis in PCa.

The prostate TME has potent immunosuppressive properties. Protumor response cytokines like IL-1α and granulocyte–macrophage colony-stimulating factor (GM-CSF), which have been shown to increase cell migration and angiogenesis, showed increased expression in the microfluidic model of the prostate TME, suggesting that this platform could be a useful tool for researching immune cell phenotypes in in vitro TME [53]. Furthermore, in Iraqi males, the IL-1β polymorphism (rs16944) increases PCa risk with aggressive behavior [54]. In a further investigation, simulated microgravity stimulated 3D development of PC-3 cancer cells and differential production of the cytokines IL-1α, and IL-1β, indicating their role in PCa cell growth and progression [55]. The above results suggest that pro-inflammatory IL-1 family members are crucial to PCa inflammation and may be targets for PCa therapy.

### IL-4 and PCa

In particular, the significance of IL-4 and its receptor, IL-4R, in promoting a prometastatic phenotype in epithelial cancer cells has been documented [56–58]. Since antibody-mediated IL-4R neutralization and IL-4Rα deletion lowered metastatic lung tumor burden and growth, IL-4R could inhibit metastatic tumor growth [57]. Furthermore, In certain circumstances, once there are low amounts of androgen, IL-4 may activate the AR [59]. IL-4 has previously been linked to the development of PCa in various studies [58, 60–63]**.**

About 7% of human genes are regulated by Yin Yang 1 (YY1), a C2H2 zinc finger nuclear transcription factor with great evolutionary conservation. YY1 is essential for controlling the inflammatory factors and tissue remodeling functions of macrophages [64]. A recent study found that YY1 was modulated by the IL-4/STAT6 pathway and that YY1 enhanced the development of macrophage-induced PCa by upregulating IL-6. Results showed that phase separation of the YY1 complex in M2 macrophages increased IL-6 expression by encouraging enhancer-promoter interactions, which accelerated the development of PCa (Fig. 3) [65]. Furthermore, tumor-promoting tumor-associated macrophages (TAMs) load TMEs. M2-like characteristics in most TAMs enhance tumor growth, immunoevasion, and metastasis. IL-4 and IL-13, which share IL-4Rα, polarize macrophages to an M2 fraction [66]. Dupilumab may change M2-like TAMs by skewing macrophages away from a protumor subtype by inhibiting IL-4Rα. These results propose targeting IL-4Rα to reduce protumor, M2-like macrophages as a cancer adjuvant. It is interesting to note that research has found that IL-4 inhibits PBMC production of the proinflammatory cytokines IL-1β, IL-6, and TNF-α [67]. As these cytokines have been reported to reduce cell viability and proliferation in LNCaP cells, reducing these cytokines may be one reason for the decrease in cytotoxicity. It can be concluded that IL-4 counteracts the cytotoxic effects of peripheral blood mononuclear cells (PBMC) on hormone-sensitive PCa cells and is involved in the immune escape of PCa.

It was demonstrated by Roca and others that IL-4-induced PCa3 cells proliferate survivin-dependently by activating the JNK pathway (Fig. 3) [61]. Moreover, based on previous research, IL-4 stimulates the phosphatidylinositol 3-kinase (PI3K)/protein kinase B (Akt) signaling pathways. The PI3K/Akt/NF-kB pathways may be accountable for the IL-4-induced AR activation in PCa [68]. IL-4’s promotion of AR signaling and PI3K/Akt and NF-kB signaling may explain the AR independence of PCa. In an androgen-deficient environment, IL-4 may signal PCa cells to survive by activating Akt and NF-kB signaling, which prevents apoptosis (Fig. 3). AR-independent PCa has elevated levels of IL-4 [68]. For advanced PCa, therapeutic strategies that target the IL-4, Akt, and NF-kB signaling systems may provide an avenue for drug development.

### IL-6 and PCa

Cytokines, particularly IL-6, are functionally involved in every stage of PCa development [25]. For instance, Han et al. [69] showed that IL-6 stimulated M2 macrophage polarization and was associated with the development of PCa cells. In addition, methyltransferase histone as a human oncogene, histone-lysine *N*-methyltransferase 2D (KMT2D) is crucial for PCa. According to recent research, KMT2D monomethylates H3K4 to increase IL-6 transcription and drive paracrine IL-6 signaling. This promotes PCa cell migration and proliferation while inhibiting PCa cell apoptosis (Fig. 3) [70]. These results imply that inhibiting KMT2D to target the IL-6 pathway and inhibit tumorigenic development may be a promising PCa therapy option. According to Binsaleh et al., PCa patients with COVID-19 had higher levels of proinflammatory cytokines (IFN-γ, TNF-α, and IL-6) than non-COVID-19 PCa patients, suggesting that these inflammatory cytokines may have caused inflammation in COVID-19 PCa patients [71]. Furthermore, a Mendelian randomization trial revealed that persistent IL-6 may raise the risk of PCa [72].

LOX-1 is a key receptor for altered low-density lipoproteins (LDLs) such as oxidized (oxLDL) and acetylated (acLDL). Recent studies show that oxLDL/LOX-1 increases reactive oxygen species (ROS), activates NF-kB, produces IL-6, and activates STAT3, enabling CRPC to be resistant to enzalutamide. These findings suggest that LOX-1/oxLDL-related new factors may enhance CRPC signaling [73]. IL-6 functions as a hub gene that may facilitate immune cell homing and differentiation in PCa [74]. In addition, gankyrin (also known as p28GANK, p28 or PSMD10), a component of the 19S regulatory cap of the 26S proteasome, has been identified by previous studies as an oncogene that contributes to oncogenesis, proliferation, drug resistance, and metastasis in multiple types of malignancies [75]. IL-6-indcued STAT3 activation leads to the establishment of Gankyrin/non-POU-domain-containing octamer-binding protein (NONO)/AR/ High mobility group box 1 protein (HMGB1)/IL-6/STAT3 positive feedback signaling network, in which STAT3 is the primary transduction molecule, and increases CRPC transformation, androgen deprivation therapy (ADT) resistance, and gankyrin expression (Fig. 3) [76, 77]. Taken together, the evidence demonstrates that gankyrin is a valid prognostic marker and treatment option for PCa patients.

Neuropeptide substance P (SP) generates proinflammatory responses via the neurokinin receptor (NK-1R) that contribute to various diseases, including cancer. Recent studies demonstrated that SP increased NF-kB target gene (IL-6) that regulate PCa cell inflammation, whereas aprepitant abolished the aforementioned effects [78]. These findings suggest that aprepitant may treat cancer-associated inflammation by modulating proinflammatory responses in PCa cells through the SP/neurokinin 1 receptor system. In addition, gastric cancer high-expressed transcript 1 (GHET1) increases PCa cell proliferation. Recent research found that the lncRNA GHET1 increased the movement of cells, their proliferation, and their resistance to paclitaxel-induced apoptosis and cell cycle arrest. GHET1 expression activates the ROS/STAT-3/Twsit1 signaling pathway, increasing IL-6 production. Knockdown of GHET1 overexpression could restrict cell movement and growth (Fig. 3) [79]. Paclitaxel resists GHET1, which may be used in clinics to treat cancer and drug resistance.

In PCa and the stromal TME, the IL-6/STAT3/Janus kinase (JAK) axis regulates angiogenesis, cell death, growth, and differentiation (Fig. 3) [80]. Based on an analysis of the function of IL-6/STAT3 in PCa, phosphorylation of STAT3 accelerates the growth of PCa and controls the pathological activity of PCa cells [81]. Overall, further clinical and preclinical research is required for the NF-kB/STAT3/JAK signaling pathways, triggered by IL-6, to be the focus of scientific research for better therapeutic strategies.

### IL-7 and PCa

Previous research has demonstrated that IL-7 influences tumor cell proliferation and transmission, lymphocyte production and differentiation in the thymus and bone marrow, and peripheral naive and memory T cell survival [82]. For instance, IL-7 increases the production of matrix metalloproteinase-3 (MMP3) and 7 and activates the Akt/NF-kB pathway to boost the movement and invasion of DU-145 PCa cells [83, 84]. Seol et al. found that overexpressing IL-7Rα in PC3 cells with lentiviral delivery increased PCa metastasis to bone in mice. In vitro, IL-7 activation promotes tumor cell mesenchymal switch, movement, and invasion via STAT5, JAK, and extracellular signal-regulated kinase (ERK) pathways, promoting epithelial–mesenchymal transition (EMT) and metastasis (Fig. 3) [85]. IL-7 may be a marker for low-recovery PCa patients. This additional information may help those who have been tested for PCa and are considering therapy determine its biological aggressiveness [86, 87].

### IL-8/CXCL8 and PCa

It has recently been demonstrated that PCa cells have higher levels of IL-8, significantly stimulating proliferation, migration, and invasion while suppressing apoptosis. IL-8 serves mechanistically by promoting the NF-kB/STAT3/Akt axis. This discovery facilitates the development of novel therapeutic approaches [88]. Previous research found that CXCL8 was strongly expressed in PCa tissues and that this expression was connected with the clinical stage. In addition, CXCL8 was shown to perform a synergistic function in both the onset and progression of PCa [89]. Similarly, IL-8 levels were significantly higher in PCa patients than in controls, suggesting that IL-8 may be a biomarker for PCa etiology and may also be an indicator for tumor progression [90]. In addition, based on investigations, upregulating CXCL8 expression via downregulating SFMBT2 stimulates the infiltration of preadipocytes and TAMs in PCa cells [91].

The most prevalent sexually transmitted parasite is called *Trichomonas vaginalis* (*Tv*). According to evidence, the growth, spread, and motility of PCa cells increased when *Tv* [Trichomonad-conditioned medium (TCM)] medium was incubated with them. The proinflammatory cytokine IL-8 also increased [92]. These findings suggest that *Tv* infection may have a role in the development of a favorable milieu promoting PCa cell growth, invasion, and inflammation. In addition, similar evidence points to the role of the CXCL8 signaling pathway in regulating the activity and expression of AR. For instance, higher levels of CXCL8 expression were associated with significantly lower AR levels and the development of an increasingly severe disease in both primary and metastatic PCa [93].

After the failure of ADT for prostatic adenocarcinomas, neuroendocrine differentiation (NED), which is connected to wingless-related integration site (Wnt) signaling activation, may be considerably observed. According to recent research, ADT increased the production of Wnt4, whereas T cell factor (TCF7L1) was activated in PCa cells and enhanced the expression of IL-8 and CXCR2 [94]. These findings imply that IL-8/CXCR2 pathway activation and disruption of AR signaling cause enhanced NED and malignancy in PCa in response to Wnt4/TCF7L1 induction. In addition, Li et al. found that CXCR2 expression is associated with PCa development and tumor grade; therefore, inhibiting it may help cure advanced therapy-resistant and metastatic PCa. The study found that NE cells express CXCR2 and CXCL8, indicating they are involved in EMT remodeling, angiogenesis, and invasion (Fig. 3) [95]. Moreover, it has been reported that IL-8 activates the mammalian target of rapamycin (mTOR) signaling pathway to protect PCa cells from GSK3-induced oxidative stress [96]. Likewise, M2 macrophages released IL-8, which supported prostate carcinogenesis through the STAT3/metastasis-associated lung adenocarcinoma transcript 1 (MALAT1) pathway [97].

### IL-9, IL-10 and IL-11 and PCa

IL-9 has unknown pro- and antitumor effects [98], and IL-9 has diverse anti- and protumor effects that involve innate and adaptive immunity [99] as well as in PCa [100]. RM-9 tumors from stressed animals expressed more IL-9 in older animals. PCa-related IL-9 production alters with age and stress, as older, nonstressed mice have much lower IL-9 levels than younger ones [101]. In addition, IL-9 is also connected to the activation and recruitment of mast cells [102, 103]. Therefore, it is probable that IL-9 stimulated mast cell activation in ApcMin/+ mice to enhance prostate carcinogenesis [104]; In fact, mast cells may play a crucial role in establishing a prostatic environment favorable to the growth of cancer [105, 106]. Furthermore, a considerable increase in the inflammatory cytokine IL-9 in mouse serum was associated with the occurrence of prostatic neoplasia [104]. Feng et al. discovered a strong association between IL-9 levels at T2 and fatigue severity from T1 to T3 [107].

In recent years, research into the mechanisms of androgen activity in PCa has contributed to a better understanding of the immunological regulatory processes played by androgens and AR in PCa patients. AR signaling has been shown to increase IL-10 and trigger receptor expressed on myeloid cell-1 (TREM-1) signaling on macrophages, consequently promoting PCa cell motility and invasion (Fig. 3) [108]. Another investigation by Faupel-Badger et al. revealed a relationship between IL-10 and PCa risk and disease severity [109]. In addition, IL-10 has been identified as a potential new biomarker for PCa in the most recent study of serum multicytokines screening [110]. P13K in the CRPC xenograft increased the expression of IL-10 in the blood, but voluntary wheel running may decrease this expression [111]. A recent study found that coculturing exosomes from PC3 cells in estrogen receptors with macrophages activated by THP-1 causes macrophages to polarize into the M2-type, which is characterized by high programmed death-ligand 1 (PD-L1) expression and IL-6 and IL-10 release [112].

In addition, TGF-1β signaling activates AP-1 and runt-related transcription factor 2 (RUNX2) transcriptional pathways. RUNX2-suppressor of mothers against decapentaplegic (Smad) and Runx2-c-Jun interact to increase IL-11 gene expression, which promotes cancer-induced osteolytic bone disease [113]. IL-11 also serves as a tumor stimulator in PCa cells and stimulates stemness [114]. Furthermore, According to evidence, LIM domain only two overexpression in prostate stromal cells, particularly peripheral zone derived stromal cells and cancer-associated fibroblasts (CAFs), may block miR-204-5p, which causes IL-11 to be upregulated [115]. Moreover, via IL-11R–STAT3 signaling, increased paracrine production of IL-11 by stromal cells in the prostate microenvironment promotes PCa cell growth and invasiveness (Fig. 3) [115].

### IL-16 and PCa

According to earlier research, the polymorphisms of the IL-16 proinflammatory gene may have an impact on the prevalence of PCa in people of African origin [116]. Likewise, according to Hughes et al., among African American males participating in PCa risk assessment, genetic diversity in the miRNA target site that encodes IL-16 may provide information about the onset of PCa diagnoses. This information may be used to develop specific PCa screening methods in the future [117]. In addition, IL-6 cytokine-related loci were strongly linked with PCa in a genome-wide association analysis [118]. In contrast, the findings of recent research from central China, none of the cytokine-related loci, including IL-16 rs7175701, and IL-16 rs11556218 were shown to be substantially linked to the development of PCa [119]. Furthermore, IL-16 is abundantly expressed in various tumor cells and favorably linked with Gleason score and clinical stage in prostatectomy tumor tissues of PCa patients. Other cancers, particularly advanced cancers, can raise blood IL-16 levels [119]. Meanwhile, the PCa screening trial examined prediagnostic IL-16 blood levels and PCa risk in 932 Caucasian patients and 942 controls [72, 120]. In Caucasians, no overall relationship between IL-16 and PCa was found [72, 120]. These results imply that there may be an ethnic variation in how these cytokine gene single nucleotide polymorphisms affect PCa in various nations, although further study is needed.

### IL-17 and PCa

There are six members in the IL-17 family: IL-17A, B, C, D, E, and F [121]. According to research, IL-17 may be a pathogenic condition that exacerbates inflammatory conditions such as chronic systemic inflammation [122]. Based on a recent study by Janiczek et al., PCa patients have higher levels of IL-17A, IL-17F, and IL-17RC expression [123]. It is suggested that IL-17 may be a reference point for PCa immunotherapy research. In addition, PCa overexpresses the inflammatory cytokine IL-17. Steiner et al. examined prostate tissues for inflammatory cytokines. Normal prostates rarely express IL-17, whereas both hyperplastic and cancerous prostates do so frequently. In cancerous prostate samples, IL-17, IL-6, and IL-8 were strongly correlated [124]. Moreover, a recent study found that IL-17F activated the PI3K/Akt signaling pathway to enhance PCa cell malignant phenotypes, indicating a potential treatment target for PCa [125]. A Galician single nucleotide polymorphism (SNP) analysis found that the IL-17 gene polymorphism −197G > A increases PCa risk [126].

PCa is caused by Epstein–Barr virus (EBV)/human papillomavirus (HPV) coinfection. Recent research found that HPV/EBV-coinfected PCa cases have higher IL-17 expression levels than noninfected cases [127]. It was suggested that the regulation of cellular behaviors caused by the HPV/EBV coinfection may be involved in the pathogenesis of PCa. PCa-associated benign prostatic hyperplasia was associated with significantly higher IL-17F expression than healthy controls [127–129]. In addition, an enzyme called cathepsin K (CTSK), a cysteine protease, reduces extracellular matrix, and is linked to osteoclast-mediated bone degeneration. The IL-17/CTSK/EMT axis controls the growth and spread of tumors and induces M2 macrophage polarization in CRPC, indicating that CTSK might be a potential target for therapy (Fig. 3) [130].

The level of glycolytic activity in PCa was associated with significant immune cell infiltration. The IL-17 signaling pathway may have a significant impact on how immune cell infiltration and tumor glycolysis interact [131]. Moreover, *Propionibacterium acnes* infection of the prostate organ may have a major impact on the infiltration of CD4^+^, IL-17^+^, and Treg cells into prostate tissue in patients with benign prostatic hyperplasia (BPH). Similarly, there is a correlation between *P. acnes* infection of the prostate organ and Treg cell infiltration in PCa patients’ prostate tissue [132]. Likewise, increased levels of CD33+ pSTAT3+ myeloid‐derived suppressor cells (MDSC), IL-17+ lymphocytes, and IL-10 mRNA expression may all contribute to the progression of BPH to PCa in PCa patients [133].

### TGF-β and PCa

It is well known that TGF-β has two roles in tumorigenesis: one as a tumor suppressor in the early stages of cancer and another as a tumor promoter in the later, metastatic stages [134]. The several stages of the PCa metastasis process have been linked to TGF-β, most notably EMT, primary tumor remodeling, angiogenesis, and the development of tumors at the metastatic location [134–136]. PCa cells and the immune system release TGF-β. TGF-β prometastasizes the extracellular environment through complex tumor cell interactions with the stromal and extracellular matrix [137]. Type I and type II serine-threonine kinase receptors of TGF-β communicate using both conventional SMAD-dependent and non-SMAD-dependent mechanisms. Zinc finger protein SNAI1 proteins, which enhance N-cadherin and vimentin and repress E-cadherin, are promoted by TGF-β, thereby promoting EMT [138, 139].

The osteoblasts epidermal growth factor receptor (EGFR)–ERK signaling pathway was activated by PCa membrane-bound TGF-α and produced prostaglandin E2 (PGE2). In a mechanism-based manner, TGF-α linked to the membrane of PCa stimulates EGFR on osteoblasts through cell-to-cell adhesion during bone metastasis (Fig. 3). In an autocrine way, the active EGFR promotes bone formation by inducing ERK signaling, prostaglandin–endoperoxide synthase (COX-2) expression, and PGE2 production [140]. It has been determined that Golgi membrane protein 1 (GOLM1), which is abundantly expressed in PCa, is a key factor for EMT in several cancers. According to a recent study, GOLM1 stimulates PCa cell line migration and invasion (Fig. 3) [141]. Furthermore, When GOLM1 is downregulated or eliminated by the p-Smad inhibitor SB431542, it promotes EMT in PCa by modulating TGF-β1/Smad2 signaling, but TGF-β1 can then reverse this effect. Through activating the TGF-β1/Smad2 signaling pathway, the essential oncogene GOLM1 promotes EMT in PCa [141]. Thus, GOLM1 may be a PCa biomarker and prognostic indicator. PCa treatment requires an effective and specific GOLM1 inhibitor.

During the extravasation stage, PCa cell migration may be significantly modulated by shear stress caused by interstitial flow [142]. It was discovered that fluid shear stress caused PC3 cells to migrate more rapidly by raising the level of αvβ3 integrins, which in turn activated downstream signaling and raised the levels of MMP-9 [142]. The unique bioreactor presented here could be applied in the future to understand the basic mechanisms underlying the proliferation and migration of various cancer types. As well, a study on GSEA discovered that it was linked to PCa carcinogenesis and the TGF-β signaling pathway [143].

Immune cell infiltration (ICI) analysis showed that PCa patients with high ICI scores had numerous TGF-β signaling pathways, which may explain immunological tolerance [144]. Patients with PCa who had more CD8+ T cells that were specific for HER-2/neu (780–788) had better progression-free survival. More CD8^+^ T cells specific for human epidermal growth factor receptor 2 (HER2)/neu were linked to lower TGF-β and IL-8 levels (780–788) [145]. According to these findings, CD8^+^ T cell immunity specific for HER-2/neu is indirectly correlated with IL-8 and TGF-levels (780–788). These findings suggest that more investigation into the link between CD8^+^ T cell immunity and HER-2/neu (780–788) with IL-8 and TGF-β levels is needed to determine the therapeutic benefit of monitoring disease progression in large patient cohorts.

### TNF-α and PCa

PCa patients had higher TNF-α level than healthy controls. PCa patients with increased inflammatory cytokines had advanced stages, metastases, and poor survival [146]. TNF-α also contributes to PCa cachexia. High TNF-α serum levels were linked to decreased reliability and higher mortality rates [147]. In addition, according to Zhou et al., PCa patients who have high serum TNF-α levels also have Gleason scores that have been upgraded [148]. According to a meta-analysis, the TNF-α-238/A polymorphism is unlikely to be a risk factor for PCa, whereas the TNF-α-308G/A polymorphism may significantly increase the risk of PCa in healthy volunteers. Further research, including considerable sample sizes, will be required in the future to elucidate the function of the TNF-α-308G/A and TNF-α-238G/A polymorphisms in prostate carcinogenesis [149]. Similarly, TNF-α-238G>A has been connected to an increased risk of prostate cancer in Indian men. They show that genes involved in inflammation (TNF-α) may accelerate the formation of PCa [150]. Through the activation of CCR7, TNF modulates the increased motility of tumor cells. A unique therapy approach for PCa patients involves focusing on TNF-α and eventually the CCL21/CCR7 axis [151].

## CXC chemokines/CXCRs and PCa

### CXCL1 and PCa

The various ways that CXC chemokines influence the initiation and development of PCa are depicted in Fig. 5. Angiogenesis-promoting ELR^+^ and CXC chemokines may boost prostate tumor aggressiveness. CXCR3 and CXCR4 moving from the cell surface to the cytoplasm may also cause protumor signaling and an alteration in aggression from moderate to severe [152]. CXC chemokines and CXCRs expression patterns varied between PCa patients depending on the metastatic site, indicating that these molecules may be involved in organ-specific PCa metastasis [153]. Both PC-EVs and HC-EVs stimulated the expression of the chemokine CXCL1 in PCF-55 cells [154]. It is also demonstrated that CXCL1 promotes angiogenesis, motility, epithelial–mesenchymal transition, adhesion, and metastasis in PCa cells [25]. In addition, through next-generation sequencing analysis, it was discovered that CXCL1 was modulated differentially during PCa [155]. As CXCL1 is important for both inflammatory and cancer-related processes, the results that have been given may help advance PCa-specific diagnostic tools.

Furthermore, bacterial infections in the microenvironment can induce persistent inflammation that not only promotes the development of prostatic intraepithelial neoplasia (PIN) and proliferative inflammatory atrophy but also accelerates the PIN process by stimulating macrophages. According to additional studies in the area where PIN develops, PIN cells can attract macrophages by secreting intercellular adhesion moleculeand CCL2, and these recruited macrophages can stimulate ERK and JNK signals in PIN cells by secreting CXCL1, which stimulates PIN cell proliferation (Fig. 5) [156]. Thus, bacterial-induced persistent prostate inflammation may be a factor in the development of PCa. Altogether, these findings provide a molecular understanding of how persistent inflammation can activate macrophages, which can accelerate PIN progression throughout PCa formation. Moreover, PCa cells stimulated by *T. vaginalis* cause inflammation through cytokine–cytokine receptor signaling pathways and CXCL1 concentration, which encourage PCa cell growth and migration [157]. These results demonstrate that *T. vaginalis* induces an inflammatory response in PCa cells in vitro and in vivo in mouse models, which accelerates the proliferation of PCa cells. Similar to how *T. vaginalis* infection causes PCa cells to become inflamed, these chemokines (CXCL1, CXCR2) cause macrophages to migrate and become activated [158].

Moreover, inflammatory bowel disease (IBD) raises the risk of PCa. According to recent research, men with IBD tend to have prostate tumors with higher T and B cell counts. Chronic colitis-affected mouse models’ prostatic CD45^+^ leukocyte infiltration increased and CXCL1, and stimulated the NF-kB and Akt signaling pathways (Fig. 5) [159]. These data show that prolonged intestinal inflammation is associated with a protumorigenic, inflammatory, and rich prostatic phenotype, which may explain how IBD males acquire PCa.

With the exception of PC3, dilated cardiomyopathy (DCM) accelerated growth, motility, and invasion in all prostate cell lines. By using fluorescence-activated cell sorting to identify CXCR2-high levels in LNCaP and 22Rv1 cells, SB225002 reduced the growth that is brought on by DCM. By using SB225002, all line migrations were decreased. In the absence of the CXCR2 inhibitor, PC3 invasion was significantly decreased [160]. These results indicated that DCM enhances PCa growth, motility, and invasion, increasing its metastatic potential. Plakophilin (PKP1) 1, an arm-repeat catenin, stabilizes cell–cell adhesion by forming desmosomes. Recently, prostate PKP1 knockdown cell lines elevated CXCL1 mRNA and protein levels. PKP1 depletion in vivo recruits immune cells to create a tumor-specific milieu. This new cancer environment may be tumor suppressive, accelerating tumor growth and progression [161].

### CXCL2 and PCa

Overexpression of CXCL2 is identified in PCa [162]. Based on the most recent data, A-kinase interacting protein 1 positively correlates with CXCL1/2 and may serve as a biomarker for PCa disease monitoring and prognosis [163]. CXC chemokines, such as CXCL2, which act as hub genes in PCa tumors, may be responsible for immune cells homing to tumors and promoting immune cell differentiation (Fig. 5) [74]. Liu et al. found that PC3 cells overexpressing tribbles homolog 1 (TRIB1) released CXCL2 and IL-8, which boosted THP-1 cell IL-12 and CD163 production. IKB-zeta ablation inhibited TRIB1-induced CXCL2 and IL-8 decreases [164].

B and T cell deficiency decreased aggressive PCa. The prostates of mice without adaptive immune systems had lower immune cell numbers and levels of the cytokines CXCL2, and TGF-β1. T and B cell deficiency protected against PCa in the Hi-Myc mouse [165]. Further research is required to determine the specific immune cell subsets that are crucial for prostate carcinogenesis, which could have an impact on the creation of innovative approaches for aggressive anticancer therapies. Moreover, research indicates that CXCL1 and CXCL2 are novel NF-kB downstream targets in PCa [166].

### CXCL3, CXCL4, and PCa

The primary factor in the tumorigenicity of PCa was the upregulation of CXCR3. It has been shown that overexpressing CXCR3 induces PCa cell migration and proliferation both in vivo and in vitro, which are connected to the development of malignancies through the ERK and Akt pathways [167]. These results imply that CXCL3 autocrine and paracrine pathways play a role in PCa formation by modulating the expression of specific genes associated with cancer progression. In addition, previous studies have shown that the CXCL3/CXCR2 axis stimulates the migration and invasion of PCa cells through the ERK signaling pathway (Fig. 5) [168]. These findings suggest that CXCL3 and CXCR2 may promote PCa progression and metastasis. In addition, in vitro and in vivo, androgen-regulated, prostate-enriched N-Myc downstream-regulated gene 3 (NDRG3) promotes PCa cell growth. NDRG3 overexpression enhances CXCL3 expression, which may accelerate tumor angiogenesis (Fig. 5) [169].

CXC chemokines such as CXCL3, which may control accelerated angiogenesis in androgen-independent PCa, were confirmed in both in vitro and xenograft models by mining the proteome of PC3-conditioned media [170]. Likewise, prostate epithelial cells prompt stromal cells to secrete proinflammatory and cancer-promoting chemokine (CXCL3). Epithelial cells also generated IL-1 cytokines that caused the phenomenon. Interactions may cause prostatic inflammation and development in early PCa [171]. Through microarray analysis, significantly increased levels of IL-6 and its receptor, IL-6R, as well as PCa immune genes such as, CXCL3 was found. These genes may lead to the development of PCa and pro-inflammatory responses [172].

Recent studies show that CXCL4/CXCR12-induced receptor tyrosine kinases promote invasive signals and metastatic proliferation. Inflammatory mediators enable CXCL12-induced PCa progenitors to adhere to the extracellular matrix. The PI3K pathway promotes prostate tumor activators that exploit CXCR4 (Fig. 5) [173]. Thus, PCa analysis will be required for future research on CXC chemokines signaling.

### CXCL5, CXCL6, and PCa

To promote “M2” macrophage polarization, TGF-β in TME altered c-Myc to boost CXCR4 expression and trigger the STAT3 pathway. When TGF is present, fibroblasts release more CXCL12, which binds to M2 macrophages’ CXCR4. In reaction to CAF interaction, M2 macrophages produced higher CXCL5, and this CXCL5 activates PCa EMT through CXCR2 and the Akt signaling pathway (Fig. 5) [174]. Sun et al. found that the inflammatory mediator CXCL5 may be a favorable prognostic factor for PCa [175]. In addition, in PCa tissues, mucosa-associated lymphoid tissue lymphoma translocation protein 1 (MALT1) is abundant. Following the nuclear translocation of NF-kB subunits (p50 and p65) by MALT1, PCa cell expressed the genes for IL-6 and CXCL5. Cell expansion, invasion, and tumor development were all induced by MALT1 (Fig. 5) [176]. Furthermore, SEPT5 downregulation enhanced tumor immune cells, notably CD8^+^ T cells. SEPT5 knockdown boosted CCL5, CXCL5, CXCL9, CXCL10, and interferon gamma receptor 1 production [177]. These results imply that SEPT5 may be a PCa prognostic biomarker and therapeutic target molecule. Moreover, the overexpression of CXCL5 regulated downstream-regulated genes such as ERK1/2, CXCL2, IL-18, and Bcl2, as well as tumor-related genes such as BAX and N-Myc. Both in vitro and in vivo, exogenous CXCL5 protein exposure or overexpression increased PCa cell malignancy [178].

**Fig. 4:**
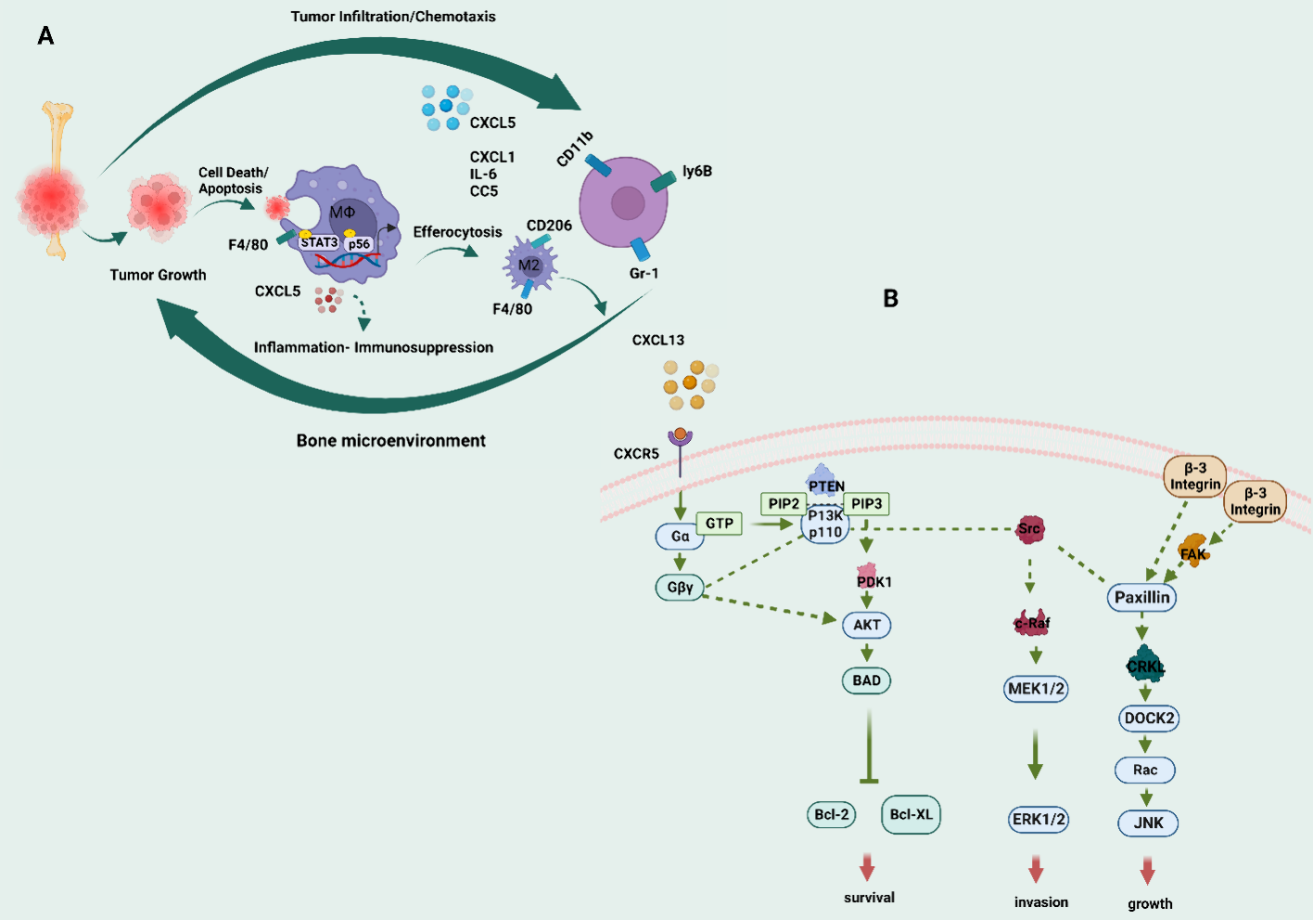
**A** Model of prostate cancer tumor growth in the bone microenvironment mediated by efferocytosis. Apoptotic cancer cells are engaged and efferocytosis by bone macrophages (MΦs), which activate Stat3 and NF-B (p65), resulting in the release of proinflammatory cytokines such as CCL5, CXCL1, IL-6, and CXCL5. CXCL5 is involved in the chemoattraction of inflammatory myeloid cells such as CD11b^+^Gr-1^+^ and Ly6B^+^ cells, as well as the promotion of M2 (F4/80^+^CD206^+^ cells) polarization, all of which lead to chronic inflammation and immunosuppression that promote tumor growth. **B** CXCL13 influences the following signaling pathways: interactions with CXCR5. CXCL13 binding to CXCR5 activates the PI3K/Akt, Raf/MEK/ERK, Integrin-3/Src/FAK, and DOCK2/Rac/JNK pathways, all of which are important in cell survival, invasion, and growth

Apoptosis-induced tumors expressed higher levels of CXCL5, a cytokine that promotes inflammation. The growth of tumors was also delayed in animals deficient in CXCL5. Patients with metastatic PCa had higher levels of CXCL5 serum in comparison to those with localized PCa or controls, and peripheral blood monocytes isolated from patients with bone metastases of prostate cancer were more efferocytic than normal controls. These data indicate that CXCL5 may be a target for cancer treatments since myeloid phagocytic elimination of apoptotic cancer cells accelerates CXCL5-mediated inflammation and tumor growth in bone (Fig. 4A) [179]. In addition, G-protein g alpha13 mediates GPCR signaling. Solid tumors, such as PCa, exhibit higher GNA13 expression. By activating G-13-NF-kB, PCa cells produce CXCL5 [180].

**Fig. 5:**
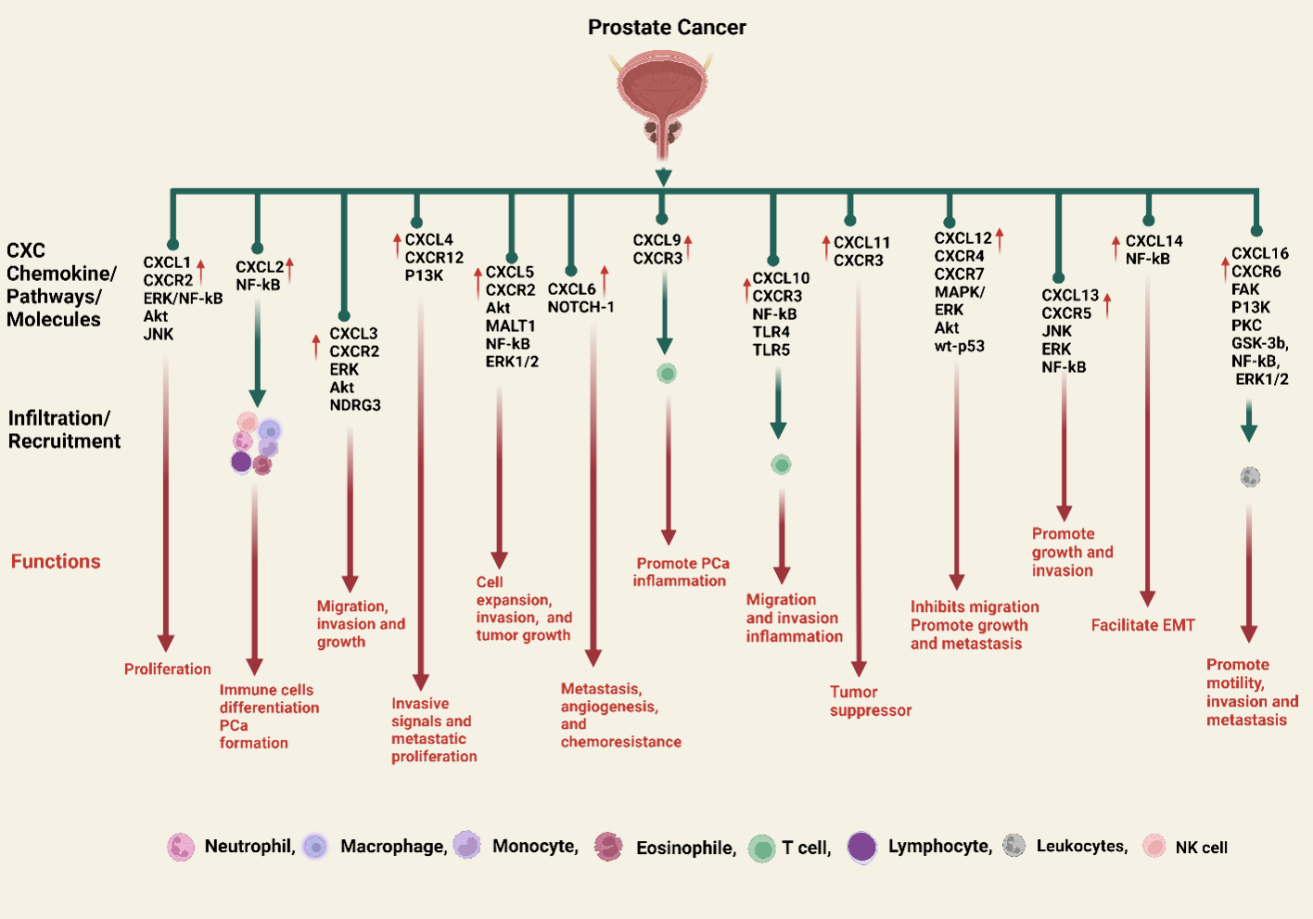
PCa development is triggered by CXC chemokines. In PCa, CXCL1 and receptor (CXCR2) induce PCa cell proliferation via ERK/NF-kB, Akt, and JNK. CXCL2 mobility primarily regulates neutrophils, monocytes, lymphocytes, macrophages, B cells, T cells, CD8^+^ T cells, NK cells, etc., to induce immune cell differentiation and, via NF-kB, PCa formation. CXCL3/CXCR2 is upregulated in PCa and contributes to PCa cell migration, invasion, and growth through the ERK and Akt pathways and the molecule NDRG3. CXCL4/CXCR12 promotes invasive signals and metastatic proliferation via the P13K pathway. CXCL5/CXCR2 via Akt, NF-kB, and ERK1/2 and the molecule MALT1 are critical for PCa cell expression, invasion, and tumor growth. High-NOTCH1 PCa expresses greater CXCL6, which induces metastasis, angiogenesis of PCa, and chemoresistance. CXCL9 attracts effector T cells, which play a key role in the inflammation and promotion of PCa cells. Likewise, CXCL10 recruits effector T cells, which also produce inflammation, and it also causes PCa cell migration and invasion through the NF-kB and TLR4/5 pathways. CXCL11 acts as a tumor suppressor in PCa. CXCL12/CXCR4/CXCR7 induce in PCa cell promote growth and metastasis via MAPK, ERK, Akt pathways while in contrast, wt-p53 pathway inhibits the CXCR4 and CXCL12 in the interactions and inhibited the migration of PCa cells to the bones. CXCL13/CXCR5 are also up-regulated during PCa via the JNK, ERK, and NF-kB pathways, leading to promote PCa cell growth and invasion. The CXCL14 facilitates the EMT in the PCa via the NF-kB pathway. CXCL16/CXCR6 recruits the leukocytes via activation by FAK, P13K, PKC, NF-kB, ERK1/2 pathways, and protein GSK-3b, which promote motility, invasion, and metastasis of PCa. The up (**↑**) arrow symbol represents upregulation

The expression of CXCL6 is elevated in PCa [181–183]. CXCL6 synthesis increases in aged prostate stroma, stimulating fibroblast and epithelial development [184], and high NOTCH1 PCa expresses greater CXCL6 [185]. These results showed that CXCL6 signaling causes PCa to grow by encouraging metastasis, angiogenesis, and chemoresistance (Fig. 5).

### CXCL9, CXCL10, CXCL11/CXCR3, and PCa

Several CXC chemokines, including CXCL9, CXCL10, and CXCL13, are present in the prostate cancer microenvironment, suggesting that these genes may aid in the prediction and interpretation of the prostate adenocarcinoma prognosis [186]. A study found that higher CXCL9 levels decreased T cell expression, which accelerated tumor growth in transgenic mice. CXCL9 overexpression reduced T cells in immunological organs and the tumor microenvironment, promoting PCa development [187]. Likewise, compared with the surrounding normal prostate, PCa tissue has a higher ability to produce T lymphocytes, which may be due to PCa cells secreting more CXCL9 during this process [188]. In laser-microdissected tissues, it was discovered that CXCL9 and CXCL11 were overexpressed and demonstrated distinction for advanced PCa [189]. According to these findings, CXCL9 upregulation was linked to the development of PCa tumors.

In addition to being a prognostic biomarker for some cancers, lysophosphatidic acid receptor 1 also contributes to PCa. According to a recent bioinformatic study, CXCL9, CXCR3, CXCL1, CXCR2, CXCL16, and CXCR6 are just a few of the CXC chemokines and receptors that are associated with lymphocytes. In PCa, CXCL9 and CXCR3 attract effector T cells (Fig. 5) [190]. Effector T cells work as inflammatory mediators [191].

According to bioinformatic analysis, PCa dramatically upregulates CXCL10 and CXCL11 [192]. In addition, metastatic PCa exhibits downregulation of Scm-like with four mbt domains 2 (SFMBT2). The role of SFMBT2 in PCa cell migration is examined. The authors claim that by upregulating CXCL8, CCL2, CXCL10, and CCL20, SFMBT2 downregulation promotes preadipocyte and TAM infiltration. When SFMBT2 is low in PCa cells, NF-kB stimulates CXCL10. SFMBT2 regulation may therefore provide a new mode of therapy (Fig. 5) [91]. Moreover, it is essential for mediating the regulation of tumor cells in tertiary lymphoid organs (TLO) that immune cells (CXCL10^+^CD3^+^T) produce CXCL10 [193]. The results suggest that the prostate has a unique environment that promotes local CXCL10 synthesis, which is crucial for attracting CXCR3^+^ effector cells to tumor-associated TLO.

In addition, CXCL10 affects binding activity and immunological responses in PCa and COVID-19 infections, respectively. CXCL10 may predict COVID-19 mortality in PCa patients [194]. In addition, three human CXCR3 splice variants—CXCR3A, CXCR3B, and CXCR3-alt—have been identified; these variants function differently in various cancer cell types [195], and CXCL9, CXCL10, and CXCL11 are ligands for CXCR3. Moreover, research has shown that PCa tissues have increased CXCR3A mRNA levels while CXCR3B mRNA levels are downregulated. Upregulation of CXCR3B, but downregulation of CXCR3A, restricts PCa cell growth and spread [196, 197]. It is therefore possible to predict that CXCL9, CXCL10, and CXCL11 may interact with CXCR3-A and activate its downstream signaling cascades, which would then decrease the expression of CXCR3-B at the posttranscriptional or posttranslational level. In light of this, the pattern of CXCR3A and CXCR3B expression in human PCa may be used as a diagnostic to determine whether the cancer is invasive. In addition, through boosting cell migration and invasion, CXCR3B downregulation may switch PCa tumor metastasis from “stop” to “go” modifying CXCR3 isoform expression influences PCa cell migration and invasion (Fig. 5) [196].

Mouse prostatic epithelial cells are stimulated by pattern recognition receptor (RRR) ligands to produce inflammatory cytokines like TNF-α, IL-6, and CXCL10, and bacterial components activate TLR4 and TLR5 signaling pathways to cause these chemokines in PECs (Fig. 5) [198]. By causing proinflammatory chemokines and cytokines to be released, TLRs can be stimulated to cause PCa inflammation. Although PKC inhibitors prevented the migration and cellular structure of CXCL10-activated PC3 cells [199].

An earlier study revealed that the PCa mouse model raised CXCL10 and CXCL11 simultaneously [200]. Moreover, Kudryavtseva et al. found that CXCL11 may be a predictive marker for PCa patients based on bioinformatic research [201]. On the other hand, a recent study found that miR-206, which targets CXCL11 as a tumor suppressor in PCa, negatively affected PCa cell growth and motility and delayed the cell cycle (Fig. 5) [202]. CXCL11 expression and CXCR3 and CXCR7 research are unknown, with a PCa hidden route investigation required. Clinicopathological and CXCL11 values were unavailable. The tumor growth molecular mechanism of CXCL11 needs further study.

### CXCL12/CXCR4/CXCR7 and PCa

CXCL12 and its receptors, CXCR4 and CXCR7, may have a role in the development of PCa metastasis and the EMT process [6], while CXCR7 function suppression may reduce the ability of organ-confined PCa to spread [203]. A recent study found that CXCL12 was substantially elevated in arterial tumor endothelial cells (TEC) and that these specific microvascular abnormalities are significant prognostic indicators [204]. The authors also mentioned the relationship between CXCR4 and CXCL12 as a potential new target to prevent tumor angiogenesis in PCa. In addition, In PCa, CAFs had upregulated CXCL12, indicating that PCa inflammation and CAF morphologies were related to PCa progression. These findings may help with PCa diagnosis and the development of alternative therapeutics [205]. Moreover, Wt-p53 inhibited the migration of PCa cells to the bones by altering the activity of CXCR4 and CXCL12 in the interactions between tumor cells and the bone marrow microenvironment [206]. These results imply that wt-p53/CXCR4 axis targeting may be a promising therapeutic approach to control PCa bone metastases.

Prior research has demonstrated that CXCR7 is more highly expressed in CRPC than in primary PCa [207, 208]. Notably, a study found that the binding of macrophage migration inhibitory factor (MIF) to CXCR7 triggers the Akt signaling pathway, promoting the growth and metastasis of CRPC tumors (Fig. 5) [208]. In addition, CXCR7 is one of the most highly upregulated genes in PCa that is administered with AR pathway inhibitors such as enzalutamide. CXCL12 and CXCR4, on the other hand, remained unchanged. According to functional analysis, CXCR7 overexpression and MAPK/ERK and/or Akt signaling are partially responsible for the ability of ARRB2 to eliminate enzalutamide resistance [207, 208]. CXCL12 and CXCR4 have a minimal impact on these effects. The findings of this study indicate that CXCR7 might be constitutively active and have a more significant impact on late-stage PCa than CXCR4, thereby providing a potentially effective therapeutic target. Likewise, via the CXCL12/CXCR4 axis, BMMSC advances PCa both in vivo and in vitro [209]. These results imply that BMMSCs may target PCa through the CXCL12/CXCR4 axis and increase PCa growth and movement both in vivo and in vitro.

Chemokines have been linked in a prominent study to treatment resistance and bone metastases in PCa through CXCR4 receptor activation. In a mechanical-logical sense, the soluble HH (SHH)/IL-6 paracrine signaling is further triggered by the bone-borne TGF-β-induced acetylated transcription factor Krüppel-like factor 5 (Ac-KLF5), thereby increasing the secretion of IL-11. The mesenchymal phenotype and tumorigenicity maintenance depend on the SHH/IL-6 pathways. It was found that Ac-KLF5 was highly expressed in the bone metastases of PCa in both in vitro and in vivo models. This suggests that PCa is resistant to docetaxel. Therefore, by focusing on KLF5 and the chemokine receptor CXCR4, the chemoresistance of PCa can be addressed (Fig. 2B) [210]. These findings support treating chemoresistant bone metastasis of PCa with Ac-KLF5/CXCR4 signaling inhibitors.

### CXCL13/CXCR5 and PCa

Based on a microarray investigation, PCa in non-neoplastic cells results in an upregulation of CXCL13 [211]. Previous research reported on the migration and invasion of CXCL13-stimulated PCa cells that were PI3Kp110-, Src-, FAK-, and DOCK2-dependent and independent, indicating the existence of cell type- and stimulus-specific conditions known to promote PCa cell migration and invasion [212]. In androgen-dependent LNCaP and PC3 cell lines, CXCR5 interacts with Gαq/11/Gβ3/Gγ9 heterotrimers and CXCR4 in the absence of CXCL13. When CXCL13 is activated, it separates G proteins from CXCR5, allowing effector molecules to be activated. CXCR5 interacts with or sequesters signals that promote Gα13 protein expression and PCa cell motility when activated by CXCL13 [213]. Furthermore, according to research, CXCL13 plays an important role in several stages of PCa development. Cluster analysis revealed a group of proteins whose activation patterns were linked to CXCL13:CXCR5 interactions in PCa. The combined activation of PI3K/Akt, Integrin-β3/Src/focal adhesion kinase (FAK), and DOCK2/JNK signaling pathways was utilized to determine their relationship with CXCL13 signaling, regulation, and integration following CXCR5 (Fig. 4B) [214]. These findings suggest that CXCL13 functions as a growth and/or cell survival factor for PCa cells and may have a role in the cellular signaling that controls PCa metastasis.

CXCL13 and CXCR5 signaling modules influence GPCR responsiveness, invasion and migration, immunological checkpoints, and innate immunity. Researchers discovered the key canonical channels and upstream regulators of CXCL13–CXCR5 expression and function. These findings point to the importance of the CXCL13–CXCR5 signaling axis in the PCa tumor immune microenvironment [215]. Moreover, CXCL13 promotes PCa cell growth via JNK signaling and invasion via ERK activation (Fig. 5) [216]. Mechanistically, Akt and ERK1/2 activation is induced by CXCL13 signaling via its corresponding receptor, CXCR5. Protein kinase C (PKC) and phospholipase C (PLC) activation may be mediated by CXCR5-coupled Gαq⁄11, which indicates that CXCL13 modulates JNK activation in LNCaP cells. However, DOCK2, a different protein that acts as an exchange factor for guanine nucleotides to activate Rac isoforms and promote cell growth, mediates JNK activation in PC3 cells. CXCL13/CXCR5, which is involved in PCa metabolism and is expressed in SPOP mutations, serves as a hub gene [217]. In addition, serum CXCL13, which also regulates PCa cell migration, integrin aggregation, and cell adhesion, is strongly linked to prostate-specific antigen and prostatic dysfunction [218]. According to Garg et al., PKCε overexpression works in conjunction with Pten deletion to stimulate PCa in mice. Together, these modifications provide prostate epithelial cells with increased capacity for motility, invasion, tumorigenesis, and growth. They also stimulate the release of CXCL13, which is regulated by the non-canonical NF-kB pathway [219]. Furthermore, androgen-responsive CXCL13 participates in androgen-induced PCa cell migration and invasion [220].

### CXCL14 and PCa

PCa purity and CD4^+^ T cells, CD8^+^ T cells, B, macrophage, dendritic, and neutrophil infiltration were associated with CXCL14 expression. The researchers additionally discovered that PCa pathology T stage and immunological score were significantly correlated with CXCL14 expression [221]. An earlier study found that CXCL14-expressing CAF and fibroblasts stimulated by CXCL14 upregulate NOS1. In CAF that expressed CXCL14, neuronal nitric oxide synthase (NOS1) was induced in response to oxidative stress, while nuclear factor erythroid 2-related factor 2 and HIF-1 transcription factor α signaling were also activated [222]. These findings identified NOS1 as an intervention target for CAF-directed cancer therapy and established critical elements of a signaling network that supports CXCL14-stimulated CAF's protumoral activities.

A tumor metastasis suppressor known as tetraspanin CD82 is known to be downregulated in a number of metastatic tumors. According to a study, CXCL14 was the gene that was downregulated the most in PrEC-31 (+CD82) compared to PrEC-31 (−CD82), while CXCL14 was highly expressed in (−CD82) [223]. The increased expression of CXCL14 in −CD82 cells may suggest a probable association between CXCL14 and CD82 in PCa carcinogenesis. In addition, CXCL14 was previously identified in an SNP analysis as a unique susceptibility gene for aggressive PCa development [224]. Furthermore, CXCL14 served as an RNA-binding protein for lncAY927529, and lncAY927529 delivered by exosomes increased CXCL14 levels in ST2 cells. Which promote PCa cell growth and invasion by regulating the bone microenvironment [225].

It has been shown that the protein CXCL14 in exosomes (sEVs) stimulates M2 macrophage polarization via the NF-kB signaling pathway, which facilitates EMT in PCa cells (Fig. 5). Although it did not impact PCa cell death, its downregulation prevented PCa cells from proliferating and invading [226]. Additionally, CXCL14 knockdown suppressed tumor growth in vivo. Collectively, exosomal CXCL14 facilitated M2 macrophage polarization via the NF-κB signaling pathway and facilitated PC advancement.

### CXCL16 and PCa

CXCL16, the only known ligand for CXCR6, is expressed in human osseous tissue, and innate human PCa cells express and have high levels of the CXCR6 protein. CXCL16 induced migration and invasion in PC3 and LNCaP cells in vitro [227]. Similar to CXCL12–CXCR4, which has been proven to take part in PCa bone metastases, CXCL16–CXCR6 is expressed in human PCa and bone specimens [227]. As a result, this study shows that CXCL16–CXCR6 plays a role in the metastasis of prostate carcinoma, even though the mechanisms by which CXCL16–CXCR6 participates in and influences the invasive patterns of PCa cells need to be investigated further. CXCL16 levels increased with ceramide levels in cohort studies, indicating that ceramides influence immunological responses in metastatic CRPC [228]. In addition, metastatic bone tissues had high CXCL16 expression. Several parameters related to CXCL16 levels in metastatic bone marrow of advanced PCa patients may predict bone metastasis. CXCR6 and CXCL16 affect tumor metastasis [229]. When exposed to IL-1 and TNF-α, prostate epithelial cells significantly increased their synthesis of CXCL16, implying that inflammatory cytokines may also be involved in CXCL16 activation. CXCL16 promoted PCa cell invasion and migration in vitro. This led to the conclusion that CXCL16 acts as a novel chemotactic factor for PCa cells via CXCR6 (Fig. 5) [230].

Mechanically, CXCL16 recruits mesenchymal stem cells (MSCs) to tumors. Tumor-derived CXCL16 interacts with MSC CXCR6 to modify MSCs into CAFs, which release significant amounts of CXCL12. CXCL12 maligns and reproduces cancer cells. EMT-prostate cancer cells express more CXCR4. CXCR4 increases metastasis [231]. Furthermore, the CXCR6–CXCL16 interaction influences cellular adhesion and motility by activating Ezrin, aggregating αvβ3 integrins, and capping the leading edge of PCa cells in an FAK/PI3K/protein kinase C (PKC)-dependent manner. The activation of CXCL16 alters cytoskeletal dynamics and promotes motility, invasion, and endothelial cell adhesion, allowing PCa cells to spread throughout the body [232]. Cancer cells’ soluble (S)-CXCL16 secretion causes a positive feedback loop, either directly through interactions with PCa cells or indirectly by creating an inflammatory milieu that promotes the growth of tumor cells [233]. Furthermore, CXCL16 from precancerous or malignant cells attracts leukocytes with CXCR6. These cells express CXCL16 and CXCR6 using TNF-α and IFN-γ. This positive feedback loop encourages inflammatory cell migration and proliferation, which promotes precancerous and malignant cell growth (Fig. 5) [234]. By phosphorylating glycogen synthase kinase-3 beta (GSK-3b), NF-kB, and ERK1/2, the CXCR6-CXCL16 axis promotes docetaxel resistance in PCa patients [235].

## Prospective theranostic therapies regarding cytokines (interleukins) and CXC chemokines

In the past few years, cancer immunotherapy has emerged as a highly effective treatment technique. A multitude of immunotherapy-based approaches, including cell-based therapies, cancer vaccines, monoclonal antibody utilization, cytokine therapy, and combination therapies, have been applied to the management of PCa. Notably, several of these methods have demonstrated promising outcomes [236]. Based on an experimental study, it was hypothesized that a combination of microtubule-disrupting drugs and the direct oncolytic and anti-angiogenic effects of the IL-12-expressing NV1042 oncolytic herpes simplex virus could enhance anti-tumor efficacy in PCa treatment [237, 238]. Prior research has predominantly demonstrated that IL-1RN can suppress the production of proinflammatory chemokines in response to TNF-α and IL-1β [239], Indicating that, apart from IL-1R1-mediated action, IL-1RN is implicated in other signal pathways. In addition, Morales and Emerson described an intralesional recombinant IFN-based trial for localized PCa. In this investigation, IFN-α was delivered directly into the prostate. This phase I trial's findings suggested that recombinant IFN-α had a positive impact [240]. In patients with CRPC, similar research was conducted with lower dosages of IFN-α-2b in combination with docetaxel [241], and the researchers concluded that in patients with CRPC, a low dosage of IFN-α2b might enhance the anticancer efficacy of docetaxel while maintaining a tolerable safety profile. Table 2 lists the most recent and comprehensive description of clinical and preclinical approaches that target cytokines, CXC chemokines, and CXCRs.

**Table 2:** Proinflammatory cytokines (interleukins) and CXC chemokine-targeted strategies in preclinical and clinical studies in PCa

Anticancer drugs/inhibitors	Target	Type of study	Outcome/result
Antagonist (IL-1RN)	CXCL1 and CXCL10	Preclinical	CXCL1 and CXCL10 are inhibited by the secret IL-1RN produced by recruited TILs [239]
Tumor-infiltrating leukocytes (TILs)/CD11b^−^	CXCL1	Preclinical	TILs/CD11b cells protect androgen-dependent cells against CXCL1 damage [260]
Lipocalin-2 (LCN2) knockout	IL-1β	Preclinical	LCN2 deficiency leads to reduced IL-1β [261]
Arbutin	IL-1β	Preclinical	Arbutin could significantly decrease IL-1β [262]
Germacranolides 1 and 2	IL-1β and IL-8	Preclinical	Germacranolides 1 and 2 reduce IL-1β and IL-8 [249]
Rye whole grain and bran products	TNF-R2	Clinical	The RP diet lowered the expression of TNF-R2 [263]
Gnetum gnemon	IL-2	Clinical	Reduce IL-2 [264]
Panax notoginseng	IL-4	Preclinical	Reduces IL-4 [265]
Resolvin D1 and D2	IL-4 and IFN-γ	Preclinical	RvD1 and RvD2 both inhibit LPS-IFNγ- and promote IL-4 [266]
Androgen deprivation therapy	IL-6 and IL-10	Preclinical	IL-10 and IL-6 were significantly lower in ADT-sensitive patients [246]
Nexrutine and exercise	IL-1β, IL-5, IL-12, and VEGF	Clinical	Both interventions reduced the concentrations of the mentioned cytokines[267]
Euterpe oleracea fruit	IL-6	Preclinical	Reduced IL-6 and induce IFN-γ [268]
GuBen-ZengGu granules	IL-6 and TNF-α	Clinical	Reduce the serum levels of IL-6 and TNF-α [269]
Docetaxel plus degarelix	IL-6 and IL-10	Clinical	Docetaxel plus degarelix reduce levels of IL-6 and IL-10 [270]
Mangiferin functionalized gold nanoparticulate	IL-6 and IL-10	Preclinical	MGF-AuNPs enhance IL-10 and IL-6 [271]
IL-15 and ADU-S100	PCa cells	Preclinical	IL-15 and ADU-S100 analogs induce the activation of NK cells and the resulting death of PCa cells [242, 272]
Cyto-IL-15	IL-15	Preclinical	Cyto-IL-15 leads to PCa growth delay [273]
6-Shogaol from dried ginger	IL-6- And TNF-α	Clinical/preclinical	6-SHO reduced IL-6 and TNF-α [274]
AgNPs of *Annona muricata*	CXCL1/CXCR2	Preclinical	AgNPs help to downregulate the CXCL1/CXCR2 axis [275]
Fucoxanthin	CXCL5, TGF-β, IL6, MMP1, TIMP1, and TIMP2	Preclinical	Fuco downregulated the expressions of the mentioned genes [276]
*Walterinnesia aegyptia* venom (WEV) + silica nanoparticles	CXCL9/10/12/13/16 CXCR3/4/5/6	Preclinical	WEV + NP decrease the mentioned CXCLs and their receptors [248]
Andrographolide	CXCL11/CXCR3/7	Preclinical	AG inhibits PCa CXCL11/CXCR3/7 by targeting cell cycle regulators [277]
chloroform	VEGF-A and CXCL-12	Preclinical	MPc inhibit VEGF-A and CXCL-12 [278]
MiR-206	CXCL11	In vivo	CXCL11-targeting MiR-206 suppresses PCa cell growth and migration [202]
*Agaricus bisporus* (white button mushroom)	IL-8 and VEGF	Preclinical	IL-8 and VEGF suppression by mushrooms may affect tumor growth [279]
Enzalutamide	IL-6 and STAT3 axis	Preclinical	Inhibition of IL-6 and STAT3 axis [250]
IL-12 expressing NV1042 oncolytic herpes simplex virus	IL-2	Clinical/preclinical	Effective method to improve antitumor efficiency in the treatment of PCa [237, 238]
Recombinant IFN-α	IFN-α	Clinical	Administered IFN-α straight into the prostate. A phase I trial found that recombinant IFN-α had a positive impact [240]
IFN-α-2b combined with docetaxel	IFN-α	Clinical	A low dose of IFN-α2b may enhance docetaxel’s anticancer properties [241]
Diosmetin	IL-6	Preclinical	Diosmetin inhibited IL-6 and IGF-1-induced PKC/Akt phosphorylation. Diosmetin may change Akt and PKCα phosphorylation, inhibiting PCa cell growth and viability [280]
Chimeric antigen receptor (CAR) T-cell therapy	IL-7	Preclinical	In xenograft models, natural killer group 2D ligand-targeting CAR T cells with IL-7 gene alteration showed improved antitumor effectiveness and longer overall survival (OS) [252]
E966-0530-45418	TGF-β	Preclinical	E966-0530-45418 reduces PCa metastasis by inhibiting CDK8 activity, TGF-β1-mediated Smad3/RNA polymerase II linker phosphorylation, and Akt/GSK3β/β-catenin signaling [281]
ZY-444	TNF signaling	Preclinical	ZY-444 targets TNFAIP3 via TNF signaling to decrease PCa growth and metastasis [282]
Receptor antagonist IL-1RA	IL-1α and IL-1β	Clinical	IL-1RA may prevent IL-1α and IL-1β activity in PCa [283]
CXCR2 blockade	TNF-α	Preclinical	CXCR2 re-educates macrophages, releases TNF-α, induces senescence, and inhibits PCa development [284]
Endothelial cell-specific molecule-1 (ESM-1) knockdown	CXCL3	Preclinical	ESM-1 inhibition decreased movement and expression of the angiogenic CXCL3 in PC-3 cells [285]
CRISPR/Cas9-mediated deletion of IL30	CXCL5	Preclinical	IL-30 deletion greatly lowers CXCL5 expression in PCa cells [286]
FAK (PF-573228) or MAPKK (PD98059)	CXCL6, IL-6, and CXCL5	Preclinical	Mesenchymal stem cells (MSC) proinflammatory cytokines/chemokine phenotypes induced by IL-1 were suppressed by FAK and MAPKK inhibitors [287]

Galustian et al. used the coculture technique to confirm that PCa cells were more capable than noncancerous cell lines (PNT2 and WPMY-1) to boost IL-15-mediated growth and cytotoxicity of NK cells [242], claiming that the activation of NK cells by the human body serves as a defensive mechanism against PCa. Furthermore, data suggest that Th2-type cytokines, such as IL-4, IL-5, IL-6, IL-10, and IL-13, might trigger antitumor responses by activating eosinophils and inducing the generation of antibodies, even though their primary roles are in host antiparasitic defense and allergy reactions. On the other hand, in cancer immunotherapy, Th1 responses may be more desirable to activate than Th2-dependent responses [243, 244]. In this regard, research using a mouse model of PCa xenografts revealed that IL-4 might promote the JNK pathway’s activation and the overexpression of survival. This anti-apoptotic protein could progress PCa [61].

Consequently, cytokines have also been demonstrated to be utilized in cancer immunotherapy; nevertheless, this approach has always had several challenges, such as beneficial dose modification and inefficient cytokine monotherapy [245]. For this reason, administering cytokines as part of cancer treatment might have serious adverse effects, similar to when the body is severely infected. Furthermore, because of the cytokines pleiotropic effects in the TME, systemic cytokine treatment has not had many therapeutic effects.

Research findings indicate that patients who responded positively to ADT had significantly lower baseline levels of specific immune markers (IL-6, IL-10, and GM-CSF). At the same time, specific pro-inflammatory cytokines (IL-5, IFN-γ, and TNF-α) exhibited significantly higher levels throughout treatment than patients resistant to ADT [246]. These studies represent significant potential for predicting treatment outcomes for patients with metastatic PCa by employing immune-related circulating biomarkers, or cytokines, to predict ADT therapy. Furthermore, silica nanoparticles containing snake venom may inhibit the growth of human PCa cells and trigger apoptosis [247], as well as significantly reduce the levels of various CXC chemokines and their receptors. Simultaneously, chemokine-driven migration lowers the prevalence of PCa [248]. Furthermore, as a cytotoxic and anti-inflammatory drug, Kłeczek et al. [249] emphasized extracts of the above-ground blooming sections of *Carpesium divaricatum*, a plant species well-known in traditional East Asian medicine. The results showed that the compound had selective cytotoxic activity against PCa cancer cell lines. By reducing the expression of IL-1β and IL-8, these extracts demonstrated a robust anti-inflammatory effect at subcytotoxic doses. The findings imply that type IV *C. divaricatum germacranolides* can have a solid anti-inflammatory impact at subcytotoxic concentrations.

The resistance of cytokines to chemotherapy, immunotherapy, and oncoprotein-targeted medications continues to be the major challenge in oncology, impacting PCa patients capability to get successful therapy over a lengthy period [236, 250, 251]. According to recent studies, an alternate tumor marker called the natural killer group 2D ligand (NKG2DL) may serve as a target for PCa patients receiving traditional chimeric antigen receptor (CAR) T cell treatment. Both in vitro and in vivo, NKG2DL-targeting CAR T cells demonstrated significantly greater cytotoxicity against PCa. On the other hand, in xenograft models, the use of CAR T cells that target NKG2DL and have had their IL-7 gene modified demonstrated improved antitumor activity and longer overall survival (OS) [252]. CAR T cell trafficking, low T cell infiltration, inadequate CAR T cell persistence, and an immunosuppressive TME are all challenges associated with the use of CAR T therapies. CAR-T cell therapy is an example of an immunotherapy that utilizes the patient’s immune system to fight the cancer; it exhibits a potent antitumor response but is inhibited by immunosuppressive factors found in the TME. Hence, continuously developing therapeutic approaches to reverse immunosuppression is required.

Based on available data, there need to be more current studies that specifically address the use of cytokines or CXC chemokines, either on their own or in conjunction with other medications or treatments. Investigating cytokines and CXC chemokines has led to promising results in preclinical, in vitro, and in vivo investigations; at this point, this area has not seen significant clinical application.

Directed immunotherapy cytokines serve a dual function in the onset and progression of PCa [251]. In circumstances of associated chronic inflammation, the formation of pro-inflammatory cytokines stimulates the infiltration of cytotoxic T lymphocytes (CTLs) into the tumor, whereas IL-1β and IL-2 cytokines boost the migration of immunosuppressive MDSCs and Treg cells [251]. In addition, immunosuppressive PD-L1 expression has been shown to be induced by TNF-α and IL-17 on the PCa cell surface, which contributes to immune resistance [253]. Immunosuppressive immune cells produce and secrete anti-inflammatory cytokines, including TGF-β and IL-10, which may suppress antitumor responses in PCa and lead to a poor prognosis [254, 255]. Consequently, circulating levels of the anti-inflammatory cytokines IL-4, IL-6, and IL-10 were higher in individuals with hormone-refractory PCa [256] and linked to elevated PSA levels. Furthermore, it has been shown that in PCa patients, the combination of TGF-β and epidermal growth factor induces tumor cells to migrate to the bone. For tumor cells to evade the immune system, TGF-β can initiate the EMT process, inhibit antitumor responses, and reduce class I major histocompatibility complex expression [257, 258]. However, inhibitory immune cells and anti-inflammatory mediators in the TME have consistently posed an enormous challenge for cancer experts. Cytokine release syndrome is a response to certain immunotherapy-based approaches, including monoclonal antibody therapy and chimeric antigen receptor (CAR) T-cell therapy. This syndrome is characterized by the aberrant synthesis and emission of inflammatory cytokines [259]. Consequently, cytokines can potentially exert a dual impact on cancer immunotherapy outcomes and immune responses.

These studies collectively lead to the conclusion that cytokines perform as a network, making identifying their role in various disease states challenging. These cytokines have dual effects on PCa: they can stimulate the immune system's anti-tumor defense while promoting tumor growth and survival by reducing anti-tumor defense or worsening inflammation.

## Conclusions and future prospectives

We attempted to shed light on the expression, underlying molecular mechanisms, origins, and critical roles of cytokines (interleukin), CXC chemokines, and CXCRs in PCa. IL-1, IL-6, IL-7/IL-7R, IL-8, IL-17, TGFs, and TNFs, as well as CXCL1–CXCL6, and CXCL8–CXCL16, all play important roles in PCa inflammatory response, metastatic spread, angiogenesis, progression, and development. Inhibiting cytokines, CXC chemokines, and CXCRs prevents the emergence and progression of PCa, which may aid in PCa detection and treatment. In contrast to the current understanding of the role of various cytokines and CXC chemokines in PCa pathogenesis, some inflammatory and immunosuppressive cytokines and CXC chemokines can promote PCa progression.

The complex network of interactions between PCa and the cytokines and CXC chemokines is currently poorly understood, which may hinder the creation of new PCa medications. The majority of cytokines and CXC chemokines are highly expressed during PCa and aid in the pathogenesis of PCa by causing inflammation, but their molecular bases have not yet been fully explored. The function of each of the inflammatory cytokines and CXC chemokines should be accurately and immediately addressed to ensure their translation into potential clinical consequences. These investigations will not only be of scientific interest, but they could also prove helpful in the future for the treatment of cancer patients.

## Data Availability

Not applicable.

## Abbreviations

PCa: Prostate cancer
ILs: Interleukins
IL-1: Interleukin-1
IL-2: Interleukin-2
IL-4: Interleukin-4
IL-5: Interleukin-5
IL-6: Interleukin-6
IL-7: Interleukin-7
IL-8: Interleukin-8
IL-9: Interleukin-9
IL-10: Interleukin-10
IL-11: Interleukin-11
IL-16: Interleukin-16
IL-17: Interleukin-17
IL-18: Interleukin-18
TGF: Transforming growth factor
TNF: Tumor necrosis factor
CXCL1: C-X-C motif chemokine ligand 1
CXCL2: C-X-C motif chemokine ligand 2
CXCL3: C-X-C motif chemokine ligand 3
CXCL4: C-X-C motif chemokine ligand 4
CXCL5: C-X-C motif chemokine ligand 5
CXCL6: C-X-C motif chemokine ligand 6
CXCL8: C-X-C motif chemokine ligand 8
CXCL9: C-X-C motif chemokine ligand 9
CXCL10: C-X-C motif chemokine ligand 10
CXCL11: C-X-C motif chemokine ligand 11
CXCL12: C-X-C motif chemokine ligand 12
CXCL13: C-X-C motif chemokine ligand 13
CXCL14: C-X-C motif chemokine ligand 14
CXCL15: C-X-C motif chemokine ligand 15
CXCL16: C-X-C motif chemokine ligand 16
PI3K: Phosphatidylinositol 3-kinase
CAFs: Cancer-associated fibroblasts
IHC: Immunohistochemistry
NF-kB: Nuclear factor-B
VEGF: Vascular endothelial growth factor
LCN2: Lipocalin-2
MCOLN2: Mucolipin-2
ERK1/2: Extracellular signal-regulated *kinase1/2*
CRPC: Castrate-resistant prostate cancers
ELF3: Epithelium-specific-like factor-3
ET-1: Endothelin 1
MAPK: Mitogen-activated protein kinase
IL-1RA: Interleukin‐1 receptor antagonist
MDSCs: Myeloid‐derived suppressor cells
PTEN: Phosphatase and TENsin
PSGR: Prostate-specific G-protein-coupled receptor
SCID: Severe combined immunodeficiency
GM-CSF: Granulocyte macrophage colony-stimulating factor
YY1: Yin Yang 1
M1: Macrophage 1
M2: Macrophage 2
TAMs: Tumor-associated macrophages
AKT: Ak strain transforming
KMT2D: Histone-lysine *N*-methyltransferase 2D
GPCR: G-protein-coupled receptor
MMP: Matrix metalloproteinase
KLF7: Krüppel‐like factor 7
oxLDL: Oxidized LDL
LncRNAs: Long noncoding RNAs
LDL: Low-density lipoprotein
ADT: Androgen deprivation therapy
NONO: Non-POU domain-containing octamer-binding protein
HMG1: High mobility group
SP: Neuropeptide substance P
NK-1R: Neurokinin receptor
GHET1: Gastric carcinoma high expressed transcript 1
ROS: Reactive oxygen species
MST2: MEN-SIN-TAN family kinases2
IFN-γ: Interferon-gamma
PKCα: Protein kinase C alpha
NKG2D: Natural killer group 2 member D
CAR T cells: Axicabtagene ciloleucel chimeric antigen receptor T cell
*Tv*: *Trichomonas vaginalis*
mTOR: Mammalian target of rapamycin
Wnt4TCF7L1: Wnt family member 4/transcription factor 7-like 1
OS: Overall survival
TIME: Tumor immune microenvironment
EMT: Epithelial–mesenchymal transition
MALAT1: Metastasis-associated lung adenocarcinoma transcript 1
ESR: Erythrocyte sedimentation rate
Ac KLF5: Acetylated Krüppel‐like factor 5
FDA: Food and Drug Administration
sCXCL16: Soluble CXCL16
ADAM100: A disintegrin and metalloprotease 100
NK cells: Natural killer cells
LMO2: LIM domain only 2
EBV: Epstein–Barr virus
HPV: Papillomavirus
MDSC: Myeloid-derived suppressor cell
BPH: Benign prostatic hyperplasia
PGE2: Prostaglandin E2
TNFAIP3: Tumor necrosis factor, alpha-induced protein 3
ELR+: Glu–Leu–Arg+
AgNPs: Green synthesized silver nanoparticles
AgNPs-F, AgNPs-L: Ethanolic extracts of fruits and leaves
CRISPR9: CRISPR-associated protein 9
AKIP1: A-kinase interacting protein 1
ESM-1: Endothelial cell-specific molecule 1
IGF-1: Insulin-like growth factor 1
MALT1: Mucosa-associated lymphoid tissue lymphoma translocation protein 1
SEPT5: Septin-5
LPAR1: Lysophosphatidic acid receptor 1
SFMBT2: Scm-like with four Mbt domains 2
TMPRSS2: Transmembrane serine protease 2
PECs: Prostatic epithelial cells
BM-MSCs: Bone marrow-derived mesenchymal stem cells
FAK: Focal adhesion kinase
TIME: Tumor immune microenvironment
NOS1: Neuronal nitric oxide synthase
IL-1RN: Interleukin-1 receptor antagonist
RP: Rye products
LPS: Lipopolysaccharide
IFN-γ: Interferon gamma
TIMP1/2: Metalloproteinases 1/2
WEV + NP: *Walterinnesia aegyptia* venom + silica nanoparticles
